# Interface‐Mediated Self‐Rectifying CsCuCl_3_ Memristors With Tunable Thresholds for Reconfigurable Neuromorphic Computing

**DOI:** 10.1002/advs.78702

**Published:** 2026-11-09

**Authors:** Qingwei Shi, Mufan Zhu, Song He, Zhen Yan, Yuezhong Lu, Shuaidi Wu, Wenju Xie, Xiaofeng Wang, Baochang Cheng, Jie Zhao

**Affiliations:** ^1^ School of Physics and Materials Nanchang University Nanchang Jiangxi China; ^2^ College of Ecology and Resources Engineering Fujian Provincial Key Laboratory of Eco‐Industrial Green Technology Wuyi University Wuyishan Fujian China

**Keywords:** halide perovskite memristor, interface engineering, reconfigurable edge computing, self‐rectifying, tunable threshold

## Abstract

We demonstrate a simple 1R self‐rectifying halide perovskite memristor with dynamically tunable thresholds for selector‐free crossbar arrays and programmable T‐ReLU mapping. The memristors achieve an ON/OFF ratio > 10^5^ and rectification ratio > 10^4^. The switching mechanism involves two synergistic processes: bulk ion migration within CsCuCl_3_ provides and sustains the low‐resistance state essential for a high ON/OFF ratio, while the Ag‐Cl‐containing oxidized interfacial layer introduces a Schottky barrier and trap filling/release mechanism that governs the HRS to LRS switching. Systematic simulations using CNN architectures reveal a dataset‐dependent threshold sensitivity: MNIST accuracy remains robust (> 98%) regardless of threshold variations, whereas CIFAR‐10 accuracy drops sharply as positive thresholds increase. Based on this complexity‐dependent trend, we exploited the bi‐directional tunability for dual‐mode applications: (i) Negative threshold operation in CNNs maintains 91.0% accuracy for low‐light image recognition (comparable to the ReLU baseline); (ii) Positive threshold modulation in a customized SparseResNet‐18 architecture is explored for sparsity‐aware edge computing, where thresholds τ ∈ [0, 0.3] are adjusted according to simulated battery levels to control the sparsity‐accuracy trade‐off. Under low‐battery conditions (τ = 0.3), the simulated network achieves 76.3% activation sparsity while maintaining 90.1% accuracy without retraining. This work highlights the potential of tunable activation thresholds for reconfigurable neuromorphic systems.

## Graphical abstract

Interface‐mediated CsCuCl_3_ memristors achieve self‐rectification and tunable thresholds, enabling selector‐free 8 × 8 arrays with > 10^5^ ON/OFF and > 10^4^ rectification. Supporting dual‐mode neuromorphic computing, negative thresholds preserve low‐light recognition accuracy (91%), while positive thresholds enable sparsity‐aware edge computing. This work advances selector‐free, sparsity‐aware artificial intelligence hardware systems.

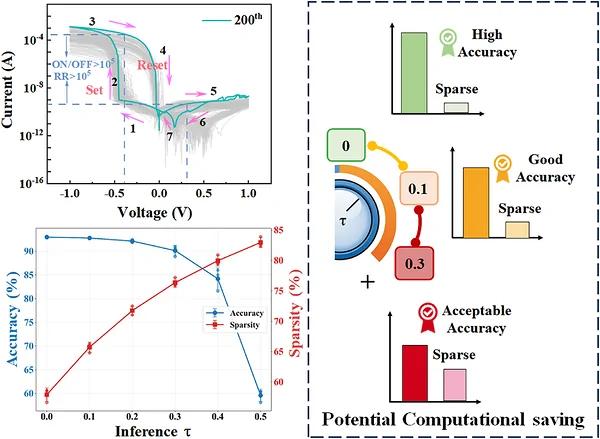

## 1 Introduction

The exponential growth of data‐intensive applications, spanning from edge intelligence to large‐scale cloud computing, has exposed the fundamental limitations of the traditional von Neumann architecture. The physical separation between the processing unit and memory results in a “memory wall”, leading to excessive data movement, high latency, and significant energy consumption [1]. To overcome these bottlenecks, neuromorphic computing, inspired by the parallelism and energy efficiency of the human brain, has emerged as a promising paradigm [2]. Among the various hardware candidates, memristors are considered the fourth fundamental circuit element and are particularly well‐suited for constructing high‐density artificial neural networks (ANNs) due to their nanoscale scalability, non‐volatility, and ability to emulate biological synaptic plasticity [3].

However, the realization of large‐scale, high‐density neuromorphic systems faces a critical challenge: the sneak‐path current issue in passive crossbar arrays. Unwanted leakage currents through neighboring cells severely degrade the read margin and limit the maximum array size [4]. While integrating a selector device (1S1R), a diode (1D1R), or a transistor (1T1R) with each memristor can mitigate this issue, it inevitably increases circuit complexity, footprint, and fabrication costs [5, 6, 7]. Consequently, self‐rectifying memristors, which inherently suppress reverse currents without additional selectors (i.e., a selector‐free 1R architecture), represent an ideal solution for achieving maximum integration density in 3D‐stackable crossbar arrays [8]. In recent years, researchers have extensively explored the implementation mechanisms of self‐rectifying memristors (SRMs) across diverse material systems, making significant strides in mitigating sneak path currents in high‐density crossbar arrays and advancing applications in In‐Memory Computing (IMC) and neuromorphic computing. For instance, Ag/CZTSSe/Mo optoelectronic memristors have demonstrated a reversible transition between bipolar resistive switching and self‐rectifying modes, with functionality dynamically tunable via light intensity and electrical bias [9]. Regarding structural optimization, Ta/HfO_x_/ZrO_y_/Pt devices based on meticulously designed oxide stacks have achieved a rectification ratio exceeding 8×10^4^ and an on/off ratio greater than 10^4^ [10]. At the array level, Miao et al. successfully implemented Sparse Matrix‐Vector Multiplication (SpMV) using Pt/HfO_2_/TaO_x_/Ta arrays, validating the practical potential of SRMs for efficient IMC with ultralow energy consumption (∼ 2.4 aJ per Set process) [11]. Furthermore, Zhang et al. demonstrated exceptional performance in TiN/HfO_x_/Pt devices, featuring a rectification ratio over 10^8^ and nonlinearity exceeding 10^5^ [4]. These devices achieved a real‐time classification accuracy of 84% on an autonomous driving dataset, a mere 0.09% deviation from the software baseline, compellingly proving the feasibility of high‐precision hardware acceleration using self‐rectifying devices.

In parallel with these developments, halide perovskites are distinguished by their unique mixed ionic‐electronic conduction and low activation energy for ion migration. For instance, Mathews et al. demonstrated self‐rectifying halide perovskite memristors via 2D/3D dimensional grading, achieving rectification ratios over 10^3^ and enabling 140 × 140 crossbar arrays for image classification tasks [12]. Lee et al. incorporated a polyvinyl alcohol (PVA) interface layer into CsPbI_3_‐based synaptic devices [13]. By leveraging O─H···I hydrogen bonding to regulate crystal orientation and ion migration pathways, they successfully mitigated interface defects and achieved highly linear and symmetric conductance modulation. Consequently, the image classification accuracy of the device in neural networks approached the theoretical limit. In the domain of optical neuromorphic computing, recent studies have embedded mixed‐halide perovskites within porous Y_2_O_3_:Eu^3+^ phosphors [14]. By exploiting interface halide migration to modulate photoluminescence intensity, these systems realized all‐optical write and read processes endowed with synaptic plasticity. Functioning as physical reservoir computing units, such systems have successfully accomplished sequence and image recognition tasks, highlighting the application potential of perovskite optical synapses in all‐optical neuromorphic computing. Furthermore, Liu et al. employed quercetin molecules to perform multi‐site chelation modulation on lead‐free FASnI_3_ perovskites [15]. This strategy suppressed Sn^2+^ oxidation and reduced defect density, yielding near‐infrared optoelectronic synapses characterized by quasi‐linear photoresponse and long decay times. Subsequently constructed 12 × 12 neuromorphic imaging arrays based on this technology demonstrated capabilities in object recognition and motion detection, underscoring the promising prospects of perovskite devices in intelligent visual systems. In our previous work, by leveraging the in situ interfacial oxidation of quasi‐2D CsPbBr_3_, we successfully engineered a dual negative differential resistance (dual‐NDR) effect [16]. This design not only enabled robust self‐rectifying characteristics within a selector‐free 1R architecture but also realized the dynamic reconfiguration of 12 distinct Boolean logic operations in a single device via voltage amplitude and polarity modulation. These attributes promise switching dynamics, enabling the construction of high‐performance, high‐density self‐rectifying memory architectures.

Despite these advances, achieving a robust self‐rectifying behavior with a high rectification ratio, high ON/OFF ratio, and controllable rectification polarity in a single perovskite device remains a materials engineering challenge [17, 18]. Moreover, current research predominantly focuses on using the memristor conductance to map synaptic weights, while the activation functions (e.g., ReLU, Sigmoid) are typically implemented by bulky external CMOS circuits [19, 20]. This separation hinders the development of fully compact “in‐memory” computing units. The capability to utilize the memristor's programmable threshold as a hardware parameter for mapping the ReLU activation function in software offers a compelling pathway to further reduce hardware overhead, yet it remains underexplored.

In this work, a high‐performance CsCuCl_3_‐based memristor is demonstrated with self‐rectification and tunable activation thresholds, achieving an ON/OFF ratio exceeding 10^5^ and rectification ratio greater than 10^4^. Through investigation of electrode‐dependent switching, interfacial chemical evolution, and charge transport dynamics, we establish that the resistive switching is governed by two synergistic processes: bulk ion migration within CsCuCl_3_ provides the low‐resistance foundation, while the AgCl_x_ interfacial layer mediates the actual switching through trap‐controlled SCLC conduction. Here, AgCl_x_ denotes an Ag‐Cl‐containing oxidized interfacial layer, without implying a specific chemical stoichiometry. Crucially, we exploit the tunable rectification characteristics to implement hardware‐software co‐design. This involves using custom CNN architectures to reveal dataset‐dependent threshold sensitivity, and subsequently exploiting this bi‐directional tunability for dual‐mode applications: negative threshold operation for low‐light recognition, and positive threshold modulation in advanced architectures for sparsity‐aware edge computing. This work highlights the potential of interface‐engineered perovskite memristors not only as high‐density storage elements but as multifunctional, reconfigurable building blocks for hardware‐based neuromorphic computing systems with potential computational savings.

## 2 Results and Discussion

### 2.1 Materials Characterization and Memristive Performance

The schematic structure of the fabricated memristive device is illustrated in Figure 1a, featuring a stacked configuration of ITO/SnO_x_/CsCuCl_3_/Ag. To optimize the film quality of the lead‐free CsCuCl_3_ active layer, an ionic liquid additive (1‐butyl‐3‐methylimidazolium tetrafluoroborate, BMIMBF_4_) was introduced into the precursor solution, coupled with an anti‐solvent engineering strategy during the spin‐coating process. As evidenced by the comparative results in Figure S1, this dual‐modification approach improved the surface morphology and coverage compared to the control sample, minimizing pinholes that could lead to premature breakdown. The cross‐sectional scanning electron microscopy (SEM) image (Figure 1b) reveals a uniform and compact CsCuCl_3_ layer with a controlled thickness of approximately 180 nm. This well‐defined thickness ensures consistent electrical pathways and reliable device‐to‐device uniformity for subsequent resistive switching measurements. The crystalline structure of the heterostructure was further investigated using x‐ray diffraction (XRD), as shown in Figure 1c. The CsCuCl_3_ film displays sharp diffraction peaks that align well with the standard hexagonal phase (ICSD PDF#82‐2273), indicating high phase purity and successful crystallization. The high‐quality crystalline structure and uniform morphology lay a solid foundation for the reliable interface‐modulated resistive switching behavior discussed in the following sections [21, 22]. The SnO_x_ layer exhibits an amorphous nature.

**FIGURE 1:**
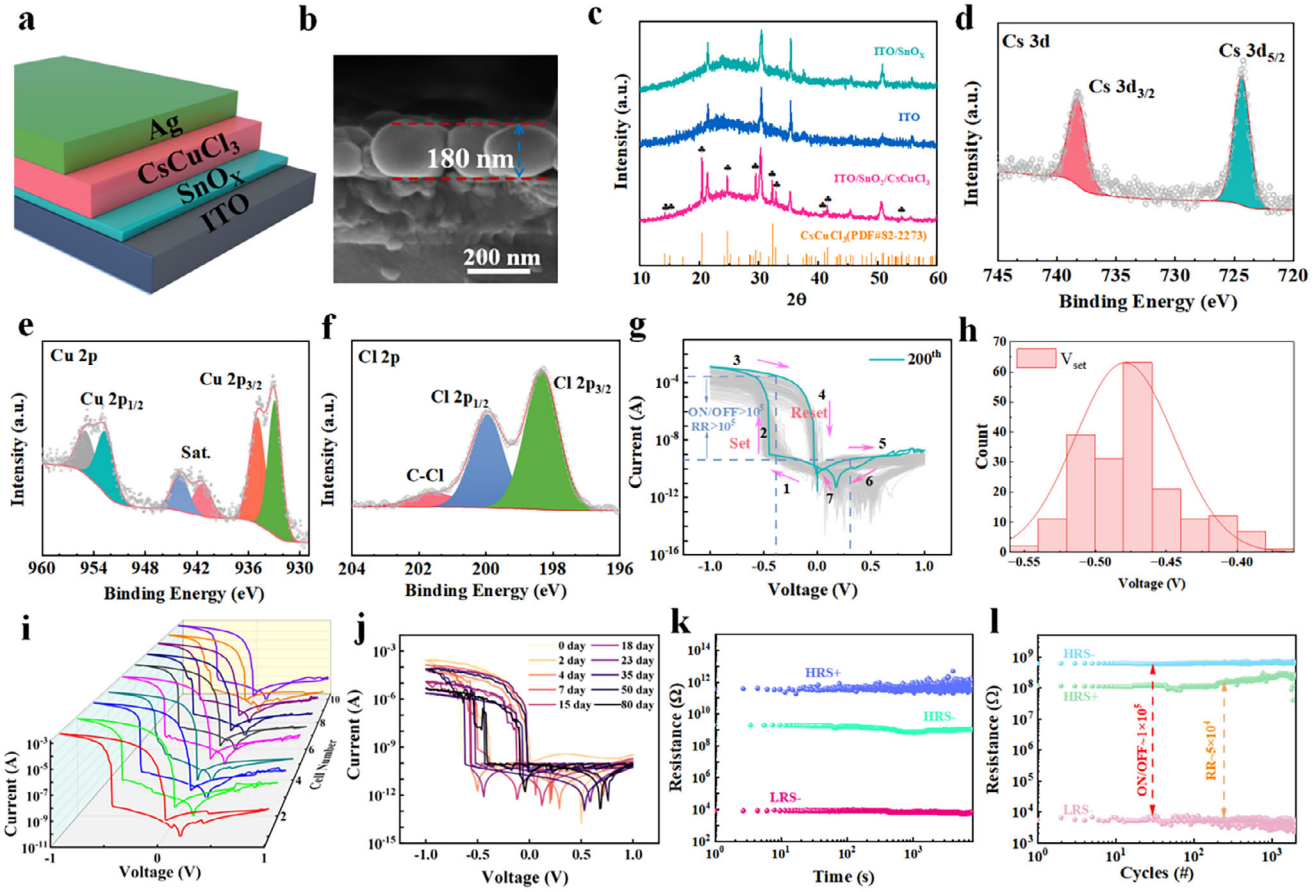
Materials characterization and memristive performance of the ITO/SnO_x_/CsCuCl_3_/Ag device. (a) Schematic illustration of the device structure. (b) Cross‐sectional SEM image showing the CsCuCl_3_ layer thickness (∼ 180 nm). (c) XRD patterns of ITO/SnO_x_/CsCuCl_3_ stacks. (d–f) High‐resolution XPS spectra of Cs 3d, Cu 2p, and Cl 2p, respectively. (g) Typical *I‐V* curves of the ITO/SnO_x_/CsCuCl_3_/Ag device measured after the initial‐state evolution (forming process), demonstrating steady‐state self‐rectifying memristive behavior over 200 consecutive cycles with the sequence 0 V→ −1 V→+1 V→ 0 V. (h) Statistical distribution of the set voltage. (i) Device‐to‐device uniformity measured from 10 independent devices. (j) Environmental stability test after 80 days of aging in ambient air. (k) Retention characteristics of HRS and LRS measured for 8000 s. (l) Endurance performance over 2000 switching cycles. The device exhibits an ON/OFF ratio > 10^5^ and a rectification ratio > 10^4^.

The surface chemical composition and elemental valence states of the CsCuCl_3_ film were investigated by x‐ray photoelectron spectroscopy (XPS), as shown in Figure 1d–f and Figure S2. The high‐resolution Cs 3d spectrum (Figure 1d) exhibits two distinct peaks at binding energies of 724.2 and 738.1 eV, which are assigned to Cs 3d_5/2_ and Cs 3d_3/2_, respectively, confirming the presence of Cs^+^ ions [7]. For the Cu 2p spectrum (Figure 1e), two main peaks located around 934.0 and 954.0 eV are observed, corresponding to Cu 2p_3/2_ and Cu 2p_1/2_, respectively. Notably, characteristic satellite peaks in the range of 940–945 eV are clearly visible, which are indicative of the Cu^2+^ oxidation state. This suggests that copper exists primarily as Cu^2+^ in the synthesized CsCuCl_3_ lattice. The presence of Cu^2+^ in the CsCuCl_3_ lattice, which is associated with the Jahn‐Teller distortion of the [CuCl_6_]^4−^ octahedra, introduces local lattice strain and a tendency toward halogen vacancy formation [23, 24, 25, 26]. This may facilitate the interfacial reactions discussed below. The Cl 2p spectrum (Figure 1f) displays two peaks at 198.4 and 199.9 eV, attributed to Cl 2p_3/2_ and Cl 2p_1/2_, respectively.

Figure 1g displays the current‐voltage (*I‐V*) characteristics of the ITO/SnO_x_/CsCuCl_3_/Ag device, revealing pronounced resistive switching (RS) behavior coupled with a significant self‐rectifying effect. Initially, the device resides in the high‐resistance state (HRS). Upon sweeping the voltage negatively, a sharp current increase occurs at a threshold voltage (*V_set_) of* approximately −0.47 V, marking the transition from HRS to the low‐resistance state (LRS). During the reverse sweep, the device resets to the HRS near 0 V, while maintaining the HRS throughout the entire positive voltage region. The *I‐V* curves exhibit an ON/OFF ratio exceeding 10^5^ and a rectification ratio (RR) greater than 10^4^, demonstrating promising switching performance and distinct unidirectional conductivity. This inherent self‐rectifying property is crucial for suppressing sneak path currents in crossbar arrays without the need for external selectors. The operational stability and uniformity of the devices were systematically evaluated. The stable self‐rectifying RS behavior was sustained over 200 consecutive *I‐V* sweeping cycles (Figure 1g), reflecting robust operational stability. Figure 1h presents the statistical distribution of *V_set_, whe*re the fitted curve peaks at ‐0.47 V, indicating a highly consistent switching threshold. Furthermore, the device‐to‐device uniformity was verified by measuring 10 independently fabricated devices (Figure 1i), all of which exhibited reproducible *I‐V* characteristics. Long‐term environmental stability was assessed by storing the unpackaged device in ambient air for 80 days (Figure 1j), confirming favorable atmospheric stability with negligible degradation in performance. Additionally, the device maintained stable HRS and LRS currents for more than 8000 s under constant bias (Figure 1k) and endured over 2000 cycles (Figure 1l). The device exhibits an ON/OFF ratio > 10^5^ and a rectification ratio > 10^4^. These comprehensive advantages suggest that the developed CsCuCl_3_‐based device holds immense potential for high‐selectivity non‐volatile memory applications with integrated rectification protection. To benchmark the performance of CsCuCl_3_ self‐rectifying memristor, we compared its retention and endurance characteristics with those of state‐of‐the‐art self‐rectifying memristors reported in recent literature (Table 1). Our memristor is comparable to or even outperforms other works, particularly among lead‐free halide perovskite‐based devices. These benchmarking results confirm that the CsCuCl_3_ self‐rectifying memristor is a promising candidate for lead‐free, high‐density selector‐free crossbar arrays.

**TABLE 1:** Comparison of the proposed ITO/SnO_x_/CsCuCl_3_/Ag self‐rectifying memristors with previous self‐rectifying memristors.

**Architectures**	**ON/OFF**	**RR**	**Threshold Tunability**	**Retention(s)**	**Endurance (cycles)**	**Refs**.
Ag/P(VDF‐TrFE):BA_2_PbI_4_/ITO	10^6^	10^6^	No	> 10^4^	200	[27]
Ag/P(VDF‐TrFE):C8‐BTBT/ITO/PEN	> 10^5^	10^5^	No	10^5^	650	[28]
Ag/Cu_3_SnS_4_‐M_O_O_3_/Mo	> 10^3^	> 10^3^	Yes	> 10^4^	200	[29]
PET/ITO/PEDOT:PSS/KPbI_3_/PMMA/Ag	10^3^	10^3^	No	10^5^	> 10^3^	[30]
Ag/CsBi_3_I_10_/ITO/Glass	10^5^	> 10^4^	No	/	10^3^	[31]
W/MAPbI_3_/FTO	> 10	> 10^2^	No	> 10^4^	150	[32]
Ti/Pt/NiO/TiO_2_/ITO	/	> 10^4^	No	> 10^3^	/	[33]
Pt/Ir/β‐Bi_2_SeO_5_/Bi_2_O_2_Se/Au	> 10^5^	> 10^5^	No	/	/	[34]
ITO/SnO_x_/CsCuCl_3_/Ag	> 10^5^	> 10^5^	Yes	8000	> 10^3^	This work

### 2.2 Interface Engineering, Switching Dynamics, and Mechanism

To elucidate the origin of the self‐rectifying memristive behavior and the specific role of each functional layer, control devices with structures of ITO/SnO_x_/Ag and ITO/CsCuCl_3_/Ag were fabricated. The corresponding *I‐V* characteristics are presented in Figure 2a. The ITO/SnO_x_/Ag device, lacking the CsCuCl_3_ active layer, exhibits a highly conductive state with negligible rectification effects. Only a minor hysteresis loop is observed in the negative voltage region, indicating insufficient memory behavior. In contrast, the ITO/CsCuCl_3_/Ag device displays memristive characteristics similar to the full ITO/SnO_x_/CsCuCl_3_/Ag structure, confirming that the switching activity is intrinsically linked to the CsCuCl_3_ layer. However, the incorporation of the amorphous SnO_x_ layer enhances the operation window in the negative voltage region and more effectively suppresses the positive leakage current. Consequently, both the ON/OFF ratio (increasing from 10^4^ to 10^5^) and the rectification ratio (*RR*) (improving from 10^3^ to 10^4^) are substantially boosted.

**FIGURE 2:**
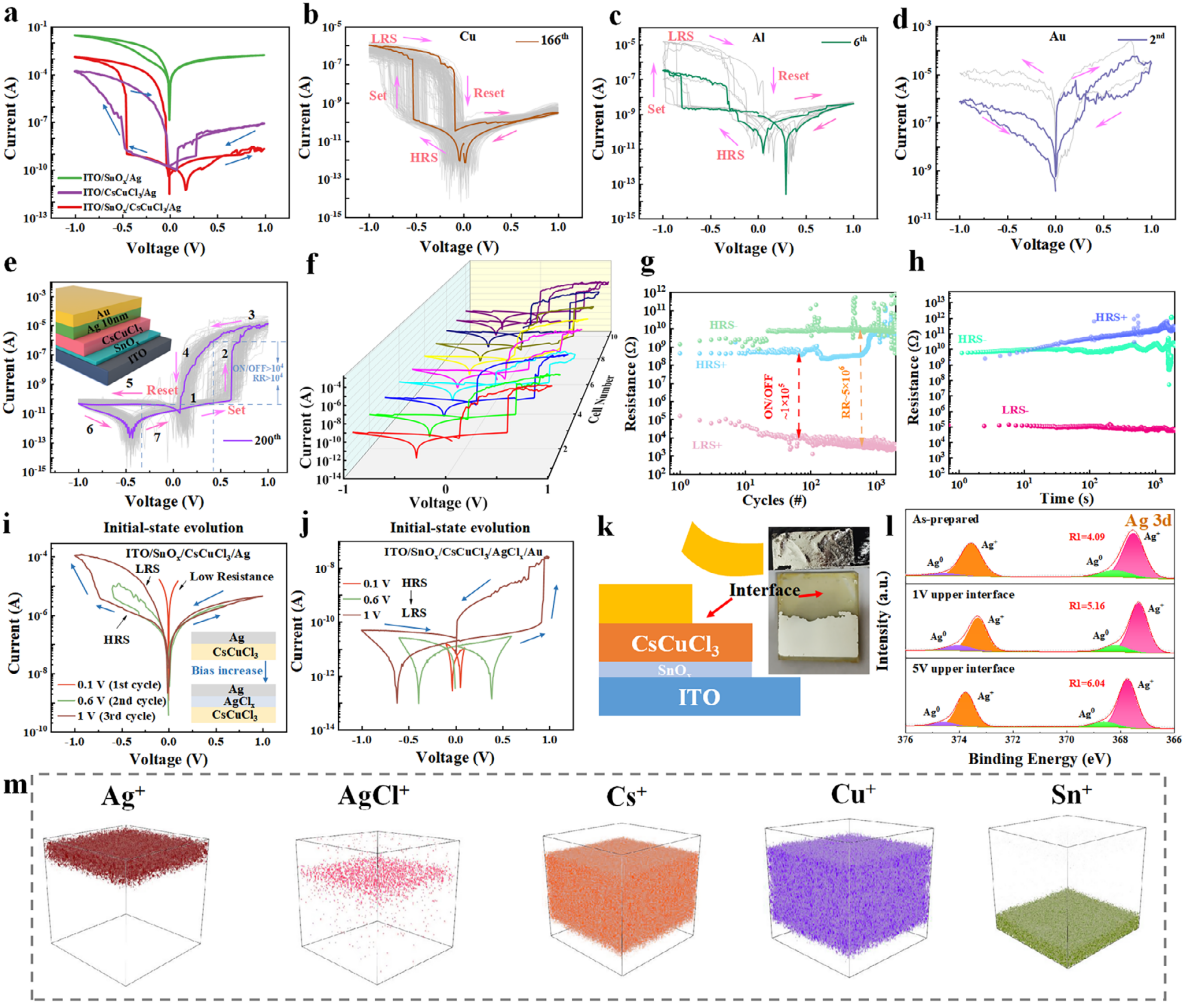
Electrode‐dependent switching characteristics and interfacial mechanism verification. (a) *I‐V* characteristics of the full ITO/SnO_x_/CsCuCl_3_/Ag device measured after the initial‐state evolution (forming process), compared with control devices (ITO/SnO_x_/Ag and ITO/CsCuCl_3_/Ag). (b–d) *I‐V* characteristics of devices with different top electrodes (Cu, Al, and Au). (e) Schematic illustration (inset) and *I‐V* curves of the AgCl_x_/Au electrode device, showing polarity reversal. (f–h) Uniformity, endurance, and retention performance of the AgCl_x_/Au device. (i, j) Initial‐state evolution (forming process) of pristine CsCuCl_3_/Ag and AgCl_x_/Au devices. The device was measured under consecutive bipolar voltage sweeps with increasing amplitudes. This evolution converts the pristine device into a memristor, which is distinct from the steady‐state switching shown in Figures 1g and 2a. (k) Schematic diagram of the electrode peeling process for XPS analysis. (l) High‐resolution Ag 3d XPS spectra of the CsCuCl_3_ interface under three conditions: pristine (0 V), after 1 V stressing (close to the normal SET/RESET voltage of ± 0.8 V), and after 5 V accelerated stressing. m) 3D ToF‐SIMS reconstructions of the ITO/SnO_x_/CsCuCl_3_/Ag stack, showing the spatial distribution of Ag^+^, AgCl^+^, Cs^+^, Cu^+^, and Sn^+^.

In our previous work, we identified that the interfacial oxidation formed between the metal electrodes (Ag and Cu) and the halide perovskite during electrical testing plays a critical role in the resistive switching characteristics. To further investigate this effect, we fabricated devices with different top electrodes (Cu, Al, and Au) on the ITO/SnO_x_/CsCuCl_3_ stack, and their *I‐V* characteristics are shown in Figure 2b–d. Consistent with our previous findings, the Cu electrode exhibits self‐rectifying memristive behavior similar to that of the Ag electrode, although the Ag‐based device demonstrates competitive cycling stability. The Al electrode, which has higher oxidizability, also shows similar switching characteristics initially but suffers from poor reproducibility; it switches to a high‐resistance state after the sixth cycle and fails to exhibit a hysteresis loop thereafter. In contrast, the device with an inert Au electrode shows slight rectification, displaying only small hysteresis loops in both positive and negative voltage regions with unstable cycling performance. These observations align with our previous discussion that the oxidizability of the metal electrode must fall within an appropriate range [16, 35, 36]. The inert Au electrode is difficult to oxidize at the interface, resulting in insignificant memristive features. Conversely, the Al electrode reacts too readily with the perovskite, forming a large interfacial barrier that leads to poor conductivity, high resistance, and unstable *I‐V* cycling. Thus, Ag and Cu serve as moderate electrode materials that balance interfacial reactivity and stability.

Interestingly, when a 10 nm Ag layer was inserted between the CsCuCl_3_ and the Au electrode (schematic inset in Figure 2e), the *I‐V* characteristics (Figure 2e) exhibited a polarity reversal compared to the pure Ag device. Specifically, the memristive loop appears in the positive voltage region, while the current is cut off in the negative voltage region. Figure 2f–h demonstrates the device's favorable uniformity, endurance, and retention (The evolution of *V_set_ is s*hown in Figure S3). This y‐axis symmetric *I‐V* behavior between the Ag and AgCl_x_/Au electrodes not only reconfirms the critical role of Ag oxidation in the self‐rectifying memristive characteristics but also reveals the modulation of the rectification direction by the electrode work function. This suggests a synergistic effect between interfacial oxidation and Schottky barrier modulation in the ITO/SnO_x_/CsCuCl_3_/Ag (or AgCl_x_/Au) devices.

To further elucidate the initial switching dynamics, we performed *I‐V* tests starting from the pristine state with progressively increasing voltage sweeps (Figure 2i,j). For the AgCl_x_/Au electrode device, the initial state exhibits a high resistance (measured at 0.1 V), and as the voltage increases, the memristive effect emerges, transitioning from HRS to LRS (measured at 1 V). In contrast, the Ag electrode device initially resides in a relatively conductive state (measured at 0.1 V). As the voltage increases, the device resistance abruptly increases before the memristive switching occurs. We attribute this transition to the electrochemical formation of an AgCl_x_ interfacial layer at the Ag/CsCuCl_3_ interface, which effectively converts the pristine conducting device into a memristor with distinct HRS and LRS states. Our previous work has shown that Ag and Cu, with their moderate electrochemical activity, are key elements for forming stable resistive switching interfaces, consistent with the observations here [16, 35]. Conversely, the AgCl_x_/Au device initially exhibits a high‐resistance state primarily due to the energy level mismatch (Schottky barrier) between the Au electrode and CsCuCl_3_. In the following sections, we will characterize and analyze these mechanisms from the perspectives of interfacial oxidation and interfacial charge transport.

It is important to distinguish between the initial‐state evolution (Figure 2i) and the steady‐state switching behavior (Figures 1g and 2a). Figure 2i shows the initial evolution of a pristine device that has not experienced any prior electrical stress. In this state, the Ag electrode has not yet formed a stable AgCl_x_ interfacial layer. The device initially exhibits a relatively conductive state due to bulk ion migration within the CsCuCl_3_ layer. As the voltage amplitude progressively increases, the electrochemical oxidation of Ag occurs gradually. A hysteresis loop begins to appear, indicating the onset of AgCl_x_ interface formation. In contrast, Figures 1g and 2a show the steady‐state switching behavior after this forming process has been completed. Once the AgCl_x_ interfacial layer is established, the device exhibits reproducible HRS‐to‐LRS SET transitions during negative voltage sweeps. Bulk ion migration within the CsCuCl_3_ layer provides and sustains the low‐resistance state throughout device operation, ensuring a high ON/OFF ratio. However, it does not participate in the switching process itself. The HRS to LRS transition is governed by trap filling and release at the AgCl_x_ interfacial layer. Thus, ion migration and interfacial trap‐controlled switching are complementary mechanisms operating synergistically: ion migration provides the low‐resistance foundation essential for a high ON/OFF ratio, while the AgCl_x_ interfacial layer provides the barrier and trap filling/release mechanism that enables resistive switching.

An electrode delamination strategy was employed to expose the buried interface for x‐ray photoelectron spectroscopy (XPS) analysis, enabling direct probing of the chemical evolution at the CsCuCl_3_/metal interface [37]. As schematically illustrated in Figure 2k, the top electrode was mechanically peeled off after electrical stressing, revealing the underlying CsCuCl_3_ surface interacting with the metal. The post‐peeling XPS analysis provides direct insight into the metal‐induced chemical changes that govern contact properties. Three states were compared to probe the interfacial chemical evolution under conditions closer to normal device operation (Figure 2l): pristine (0 V), after 1 V stressing (close to the normal SET/RESET voltage of ± 0.8 V), and after 5 V accelerated stressing. The Ag^+^/Ag^0^ area ratios are 4.09 at 0 V, 5.16 at 1 V, and 6.04 at 5 V. The increase from 0 to 1 V (ΔR = 1.07, about 26%) indicates that Ag oxidation begins under realistic switching biases and is already significant at voltages close to the normal SET/RESET voltage. The further increase from 1 to 5 V (ΔR = 0.88) shows that accelerated stressing amplifies the interfacial oxidation. The Cl 2p spectra do not show a clear shift between 0 and 1 V, possibly because the Cl chemical environment changes only slightly under normal operation or because XPS is less sensitive to the Cl chemical shift in this system. After 5 V stressing, however, the Cl 2p peaks shift toward lower binding energy (Figure S4a), consistent with the formation of Ag‐Cl bonding.

The interfacial chemical changes were amplified for XPS detection by applying 5 V accelerated stressing and comparing the Ag and Au electrodes. The corresponding XPS spectra are shown in Figure S4b–d. Figure S4b displays the high‐resolution Ag 3d core‐level spectra, where the peaks located at 367.7 and 373.8 eV are assigned to the Ag 3d_5/2_ and Ag 3d_3/2_ orbitals of oxidized species (Ag_2_O/AgCl), respectively. The peaks at 368.3 and 374.2 eV correspond to the metallic Ag^0^ state. To quantify the extent of interfacial oxidation, we calculated the intensity ratio (*R*) of the oxidized Ag^+^ (Ag 3d_5/2_) to the metallic Ag^0^ (Ag 3d_5/2_), defined as *R* = Area_Ag+_/Area_Ag0_. At the CsCuCl_3_/Ag interface, the *R* value increases from 2.22 (pristine) to 3.10 after electrical stressing. This marked enhancement indicates that the applied electric field actively promotes the oxidation of Ag atoms, leading to an accumulation of oxidized products (Ag_2_O/AgCl) at the interface [37]. In stark contrast, the Au electrode exhibits negligible variation in its chemical state before and after bias treatment (Figure S4c), confirming the chemical inertness of Au under operating conditions. Furthermore, the initial Cu 2p spectrum, with its characteristic Cu^2+^ peaks and satellites, serves as a baseline. The enhancement of the 934.5 eV (Cu^2+^ main peak) intensity relative to the 932.4 eV component after Ag electrode removal signals a substantial chemical alteration at the interface. This is consistent with Ag's ability to extract Cl from the perovskite lattice due to the favorable formation energy of AgCl_x_. The resultant chlorine vacancies are expected to be partially compensated by oxygen from the ambient environment, a mechanism well documented in halide perovskites [38, 39]. The enhanced Cu 2p signal is interpreted as a chemical consequence of Cl extraction from the CsCuCl_3_ lattice, rather than as the direct cause of the resistive switching. The detailed mechanism governing the switching process, including the role of the interfacial AgCl_x_ layer, will be elaborated in the following sections through electrical transport analysis and temperature‐dependent measurements. For the Au‐contacted sample, the minimal change in the Cu peak ratio reflects its inertness, leaving the original halide surface essentially intact. The Cl 2p core‐level spectra (Figure S4d,e) show distinct peaks for Cl 2p_3/2_ and Cl 2p_1/2_ with no observable binding energy shift upon electrical stressing for either Ag or Au electrodes. The full XPS survey spectra are shown in Figure S5.

As a further confirmation of the formation of AgCl_x_ at the Ag/CsCuCl_3_ interface, we performed time‐of‐flight secondary ion mass spectrometry (ToF‐SIMS) depth‐profiling on the ITO/SnO_x_/CsCuCl_3_/Ag structure over a 300 µm × 300 µm area (Figure 2m and Figure S6). The depth profiles reveal a clear sequential signal evolution from the top electrode to the substrate, with the outermost Ag layer, the CsCuCl_3_ perovskite layer, and the underlying SnO_x_/ITO layer being detected. Notably, a distinct AgCl^+^ signal is observed at the upper interface region between the Ag layer and the CsCuCl_3_ layer, directly evidencing the formation of AgCl_x_‐containing species at the interface. This result is consistent with the XPS binding energy analysis (Figure 2l), which shows characteristic Ag‐Cl bonding features at the same interface. Collectively, these results corroborate the proposed mechanism that electrically driven interfacial oxidation of the active metal electrode (Ag) is a key factor in modulating the Schottky barrier and enabling the observed memristive behavior.

With the aim of elucidating the role of the interface in the memristive charge transport behavior, we systematically analyzed the energy band alignment of the device stack. Figure 3a presents the UV–vis absorption spectrum of the CsCuCl_3_ film. The optical bandgap (E_g_) is determined to be 2.61 eV via the T_auc_ plot analysis (inset, Figure 3a). Furthermore, Ultraviolet Photoelectron Spectroscopy (UPS) was employed to determine the valence band structure and work function. As shown in Figure 3b,c, the valence band maximum (VBM) is located at 3.81 eV below the Fermi level, and the work function is measured to be 3.31 eV. Based on these parameters, the complete energy band diagram of the ITO/SnO_x_/CsCuCl_3_/metal device is constructed in Figure 3d. The distinct roles of bulk ion migration (bulk), AgCl_x_ formation (interface), and electronic trapping (AgCl_x_ layer) are schematically illustrated in Figure 3e (left). Bulk ion migration within the CsCuCl_3_ layer provides the low‐resistance state of the pristine device and sustains it throughout operation, ensuring a high ON/OFF ratio. The electrochemical formation of the AgCl_x_ interfacial layer, involving the redistribution of Ag^+^ and Cl^−^ species at the interface, occurs during the initial forming stage. This one‐time interfacial reconstruction establishes the Schottky barrier and introduces trap states that govern the self‐rectifying behavior. Electronic trapping and detrapping within the reconstructed AgCl_x_ interface govern the reversible HRS to LRS switching. The filling of traps triggers the HRS to LRS transition, while the subsequent release of trapped charges drives the LRS to HRS transition. Bulk ion migration provides the low‐resistance foundation, AgCl_x_ formation establishes the rectifying barrier, and electronic trap dynamics control the switching transition. Correlating the band alignment with the rectification characteristics, we can identify the dominant carrier transport mechanisms. For the device with a low work function Ag electrode, the energy barrier for electron injection is lower than that for holes, resulting in electron‐transport‐dominant behavior with conduction in the negative voltage region and cut‐off in the positive region, as schematically illustrated in Figure 3e (right). Conversely, the device with a high work function AgCl_x_/Au electrode favors hole injection, resulting in cut‐off in the negative voltage region and conduction in the positive region (Figure S8).

**FIGURE 3:**
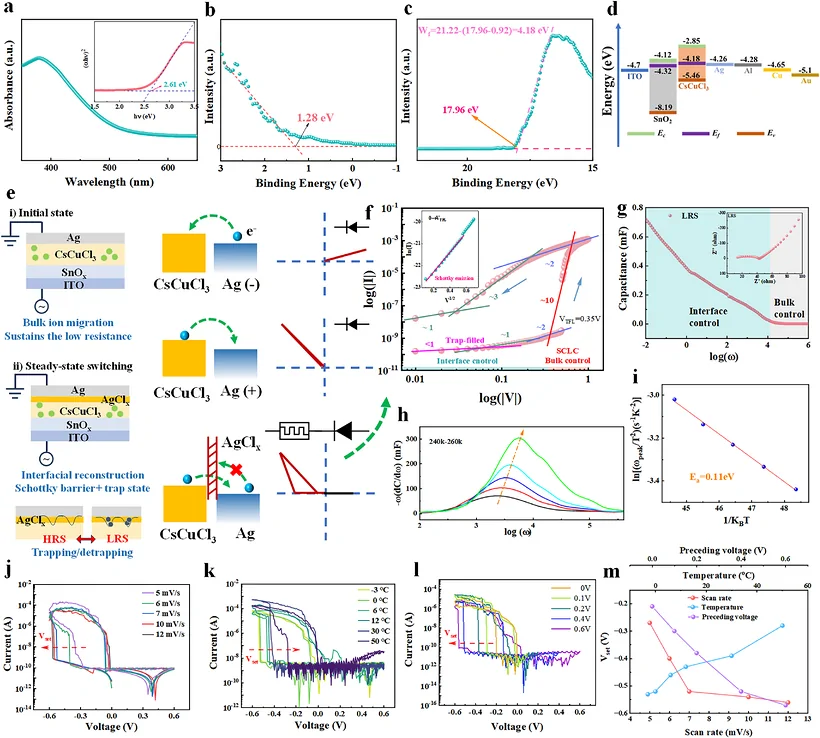
Energy band alignment, conduction mechanism, and switching dynamics. (a) UV–vis absorption spectrum of CsCuCl_3_ with T_auc_ plot inset. (b, c) UPS spectra showing the valence band onset and secondary electron cutoff. (d) Energy band diagram of the ITO/SnO_x_/CsCuCl_3_/metal device. (e) Schematic illustration of the switching mechanism for the Ag‐electrode device. Left: (i) bulk ion migration within the CsCuCl_3_ bulk provides the initial LRS; (ii) the formation of interfacial AgCl_x_ gives rise to Schottky barriers and trap states that govern self‐rectification; electronic trapping and detrapping inside the AgCl_x_ layer control reversible HRS‐to‐LRS switching. Right: carrier transport mechanism for the Ag‐electrode device. (f) Double‐logarithmic *I‐V* plots showing the SCLC fitting regions. Inset: Schottky emission fitting. (g) Capacitance‐frequency (C‐f) characteristics showing giant low‐frequency capacitance. Inset: Nyquist plot. (h) Temperature‐dependent dielectric relaxation peaks. (i) Arrhenius plot of the peak frequency yielding an activation energy of 0.11 eV. (j) Frequency dependence of *V_set_*. (k) Temperature dependence of *V_set_* from 3°C to 50°C. (l) *I‐V* curves showing the modulation of negative V_set_ by preceding positive voltages of different amplitudes. (m) Summary of V_set_ variation with frequency, temperature, and preceding positive voltage amplitude.

We analyzed the double‐logarithmic *I‐V* plots of the resistive switching region, as shown in Figure 3f, to elucidate the charge transport mechanism and the origin of the memory effect [40]. In the low bias region, the fitting slope n < 1 indicates that carrier injection is limited by the interfacial Schottky barrier formed by the oxidized AgCl_x_ layer. This is further corroborated by the ln(I)∝V^1/2^ fitting in the inset of Figure 3f, which confirms Schottky emission as the dominant injection mechanism at low biases. As the voltage increases, the device enters the Ohmic region (n ≈ 1), where the trap states within the AgCl_x_ reach thermal equilibrium, and the free carrier concentration remains constant, leading to a linear current increase. When the voltage exceeds a critical threshold (*V_set_), th*e slope sharply rises to n > 2, marking the transition to the trap‐controlled SCLC regime [41]. In this region, all available trap states are filled, and newly injected carriers contribute directly to conduction, triggering a steep current increase. Notably, the conduction mechanism exhibits significant asymmetric evolution during the forward and reverse voltage sweeps, revealing the memory characteristics of trap dynamics. During the return sweep, the slope follows a path of n > 2, n ≈ 2, and n ≈ 1. The emergence of the n ≈ 2 plateau corresponds to ideal SCLC behavior. This indicates that during voltage reduction, the traps remain filled and do not release immediately, maintaining a high‐conduction channel. This discrepancy between the filling path (n > 2) and the holding path (n ≈ 2) constitutes the hysteresis loop in the *I‐V* curve, providing evidence that the non‐volatile memory originates from the stable retention of trapped charges within the interfacial AgCl_x_ layer. Collectively, these results establish a unified model: the electrode/AgCl_x_ interface modulates carrier injection (Schottky barrier), while the trap filling and retention within the AgCl_x_ layer govern the resistive switching memory.

The switching mechanism was elucidated from both ionic and electronic perspectives by impedance spectroscopy analysis. This encompasses the mobility of charge carriers within the perovskite layer, charge recombination processes, and the material's inherent dielectric properties [42]. The low‐frequency capacitance (∼ 0.7 mF) observed in halide perovskite devices has been extensively reported and attributed to multiple synergistic mechanisms, including ion‐migration‐driven interfacial electrical double‐layer polarization, stoichiometric polarization, interfacial charge trap effects, and electrode polarization [43, 44, 45]. At high frequencies, the interfacial charges cannot respond to the alternating electric field, causing the capacitance to drop to the intrinsic bulk level [46]. The Nyquist plot (inset of Figure 3g) exhibits a low‐frequency arc characteristic of interfacial space charge accumulation, consistent with the giant low‐frequency capacitance observed in the C‐f characteristics [47].

Furthermore, we analyzed the dielectric relaxation behavior in the temperature‐dependent capacitance spectra in the LRS. As shown in Figure 3h, distinct relaxation peaks are observed. With increasing temperature (240–260 K), the peaks shift toward higher frequencies, indicating that the interfacial charge relaxation process is thermally assisted and accelerated. Through Arrhenius fitting (Figure 3i), the activation energy (E_a_) for this relaxation process is calculated to be 0.11 eV. The relationship is expressed as:

(1) $$\omega_{\mathrm{peak}}=\beta T^{2}\;\exp\;\left(-E_{a}/\mathrm{kT}\right)$$

In this equation, 𝛽 serves as a temperature‐dependent prefactor [48]. This extremely small activation energy provides critical insights into the conduction mechanism. It rules out the possibilities of intrinsic band‐to‐band transition (typically > 1 eV) and deep‐level trap hopping (typically > 0.3 eV) [49]. We attribute the observed activation energy to an ion‐electron coupled relaxation process: (i) halide ion migration within the interfacial AgCl_x_ layer and CsCuCl_3_ bulk, and (ii) thermally activated electron trapping/detrapping at shallow defect states introduced by the interfacial AgCl_x_ phase [43, 50, 51]. The intrinsic low barrier for Cl^−^ migration in both the disordered AgCl_x_ layer (∼ 0.09–0.12 eV) and the CsCuCl_3_ bulk (∼ 0.08–0.25 eV) enables their synergistic contribution, while ion migration and electron trapping/detrapping are kinetically coupled through local lattice rearrangements, yielding an effective single activation energy [52, 53, 54, 55, 56, 57, 58]. By correlating the impedance results with the initial‐state evolution in Figure 2i, we distinguished the respective roles of ion migration and electronic trapping in the resistive switching mechanism. Bulk ion migration within CsCuCl_3_ provides the initial low‐resistance state of the pristine device. As the voltage progressively increases, the AgCl_x_ interfacial layer forms and introduces trap states that govern the subsequent resistive switching. This interpretation is consistent with the SCLC analysis (trap‐filled limit behavior, n > 2) and the negative temperature coefficient of *V_set_* (thermally assisted trap ionization). Thus, the ionic and electronic processes are not competing but complementary, operating at different stages of device operation: ion migration establishes the initial state, while interfacial traps dominate the switching behavior.

The dependence of Vset on voltage amplitude, temperature, and scanning frequency was examined to separate the contributions of ion migration and electronic trapping to the resistive switching mechanism (Figure 3j–m). Figure 3j shows that *V_set_ *increases by approximately 0.37 V with increasing scanning frequency. This frequency dependence, in addition to its contribution from ion migration, also arises from the finite relaxation time required for interface charge accumulation and subsequent barrier modulation [59, 60, 61]. The increase of *V_set_* with increasing sweep amplitude reflects voltage‐controlled trap filling depth within the AgCl_x_ interlayer. Under higher sweep voltage, the applied bias fills deeper trap states with longer emission time constants. These deeply filled traps remain partially occupied during the reverse sweep, leaving residual trapped charge that elevates the threshold for subsequent trap saturation, thus increasing *V_set_* [62, 63]. To comprehensively evaluate the thermal effect, we measured *I‐V* characteristics over a wide temperature range from 3°C to 50°C. As shown in Figure 3k, *V_set_* exhibits a monotonic decrease with increasing temperature, with a total drop of 0.32 V. This significant negative temperature coefficient confirms that the Set process is a strongly thermally activated process. This is attributed to two aspects: First, elevated temperature enhances thermionic emission at the interface, allowing carriers to surmount the Schottky barrier at lower voltages. Second, thermal energy assists in the ionization of carriers from shallow traps, effectively reducing the effective trap density, thereby lowering the trap‐filled limit voltage (*V_TFL_*) [64, 65]. This stable trend over a wide temperature range is not only highly consistent with the extracted low activation energy (0.11 eV) but also rules out unstable mechanisms such as thermal breakdown [41, 64, 65].

Direct probing of the interfacial electronic structure modulation induced by different electrode materials was achieved by KPFM measurements on two half‐covered samples: one with Ag and another with AgCl_x_/Au deposited on half of the CsCuCl_3_ surface (with the other half left bare as reference), as shown in Figure S9a–d. The bare CsCuCl_3_ surface exhibits a CPD of approximately −100 mV. On the Ag‐deposited sample, the Ag‐covered region shifts to approximately −130 mV, corresponding to an increase in surface work function of about 30 meV. In contrast, on the AgCl_x_/Au‐deposited sample, the AgCl_x_/Au‐covered region shifts to approximately −60 mV, corresponding to a decrease in surface work function of about 40 meV. These KPFM results provide direct experimental evidence that the electrode material and interfacial chemistry govern the switching polarity through modulation of the interfacial barrier.

For quantitative extraction of the barrier height, we performed temperature‐dependent *I‐V* measurements from 50°C to 75°C and analyzed the forward‐bias characteristics based on the thermionic emission (TE) model (Figure S9e,f, Tables S1, and S2) [66, 67, 68]. The ideality factor n remains close to unity (1.03–0.95), confirming TE‐dominated transport. The Richardson plot (ln(I_0_/T^2^) vs. 1000/T) exhibits favorable linearity (R^2^ = 0.993), yielding an effective Schottky barrier height of approximately 0.46 eV, consistent with the temperature‐dependent V_set_ behavior (Figure 3k). The activation energy extracted from the LRS (0.11 eV) is significantly smaller than this barrier height, indicating that LRS transport is governed by trap‐assisted tunneling or hopping rather than direct thermionic emission over the entire barrier. These results establish that the interfacial Schottky barrier (∼ 0.46 eV) governs carrier injection and the switching threshold in the HRS, while LRS transport proceeds through a low‐activation‐energy (0.11 eV) trap‐assisted process. The discrepancy between the KPFM surface signal (∼ 40 meV) and the extracted barrier height (∼ 0.46 eV) can be understood through the interface dipole effect. At the metal/CsCuCl_3_ interface, the AgCl_x_ layer introduces an interfacial dipole that modulates the vacuum level alignment and the effective Schottky barrier height. The KPFM signal reflects only the residual field of this dipole layer outside the surface, which is substantially smaller than the actual potential drop across the interface, consistent with the rapid decay of dipole fields outside the sample surface [69, 70, 71]. Thus, the barrier height extracted from KPFM is smaller than that obtained from thermionic emission analysis. Furthermore, the AgCl_x_ layer and the Au capping layer play distinct but synergistic roles. The Au‐only device (Figure 2d) exhibits only weak rectification without significant memristive behavior, confirming that the AgCl_x_ layer is essential for the memory effect. Both the Ag‐only and AgCl_x_/Au devices contain the AgCl_x_ layer but exhibit opposite switching polarities (Figure 2a,e), indicating that AgCl_x_ provides the interfacial dipole and trap states, while the Au work function modulates the dominant carrier injection direction. This synergistic effect accounts for the polarity reversal observed in our devices.

Based on these observations, bulk ion migration and interfacial electronic trapping play distinct but complementary roles. Bulk ion migration within CsCuCl_3_ provides the initial low‐resistance state, while the AgCl_x_ interfacial layer introduces trap states that govern the subsequent switching. The SCLC analysis reveals trap‐filled limit behavior (n > 2), and the negative temperature coefficient of *V_set_* is consistent with thermally assisted trap ionization. The frequency and amplitude dependences of Vset further reflect interface charge accumulation and voltage‐controlled trap filling depth within the AgCl_x_ interlayer. KPFM measurements directly confirm that Ag and AgCl_x_/Au contacts produce opposite surface work function shifts (+30 meV vs. −40 meV), establishing that interfacial chemistry governs the switching polarity through barrier modulation. The extracted barrier height of ∼ 0.46 eV from thermionic emission analysis supports that HRS transport is governed by the interfacial Schottky barrier while LRS transport proceeds through trap‐assisted processes. The framework established by Chen et al. further supports this interpretation, where controlled introduction of trap states at the interface through Cs_2_CO_3_ deposition regulates resistive switching, analogous to the role of AgCl_x_ layer in our devices [72]. Thus, ion migration and electronic trapping are not competing but complementary: ion migration provides the initial LRS, while the AgCl_x_ interfacial layer provides the barrier and trap filling/release mechanism that governs resistive switching.

In addition to temperature‐ and frequency‐dependent threshold modulation, we demonstrate that the negative threshold voltage (*V_set_*) can be electrically programmed by applying a preceding positive bias. As shown in Figure 3l,m, the magnitude of V_set_ increases monotonically from approximately −0.2 to −0.6 V as the amplitude of the preceding positive voltage increases from 0 to 0.6 V. The preceding positive bias alters the occupancy of interfacial traps at the AgCl_x_/CsCuCl_3_ interface. This modulation of the charge state changes the effective barrier height, which in turn determines the negative threshold voltage (V_set_) required for subsequent switching. This interpretation is consistent with our proposed unified mechanism, where the AgCl_x_ interfacial layer modulates carrier injection through a Schottky‐type barrier, while trap filling and release govern the resistive switching memory. The programmed threshold can be retained as a nonvolatile state. As shown in Figure S10, programming the device with different preceding positive biases (0, 0.1, 0.2, 0.4, and 0.6 V) yields corresponding *V_set_* values of −0.2, −0.3, −0.4, −0.5, and −0.6 V. When the applied negative voltage exceeds the programmed *V_set_*, the device switches from the HRS to the LRS with an ON/OFF ratio exceeding 10^5^. The resulting LRS remains stable for over 1000 s with minimal drift. Figure S10 also presents the cycle‐to‐cycle variability, device‐to‐device reproducibility, and programming endurance of the programmed threshold states. These results confirm that the threshold states are nonvolatile and sufficiently reliable for the proposed T‐ReLU parameter mapping.

### 2.3 Crossbar Array Demonstration and Neuromorphic Computing With Tunable Thresholds

To assess the efficacy of leakage current suppression within a high‐density architecture, 8 × 8 crossbar arrays comprising both Ag and AgCl_x_/Au memristors were fabricated, as depicted in the optical image in Figure S11. Figure 4a,b illustrates the *I‐V* characteristics of single cells within the array for both Ag and AgCl_x_/Au electrode devices, demonstrating their ability to suppress reverse leakage currents. For the Ag‐based device, after being SET at a negative voltage (‐0.8 V), the cell switches from HRS to LRS. Crucially, when the read voltage is switched from −0.3 V (LRS) to +0.3 V, the device exhibits HRS, indicating effective current cut‐off in the reverse bias direction. This rectifying behavior provides a built‐in sneak path suppression mechanism, essential for preventing crosstalk in passive arrays. Conversely, the AgCl_x_/Au device exhibits the opposite polarity: it is SET at a positive voltage, conducting at +0.3 V (LRS) and cutting off at −0.3 V (HRS). The uniformity and reliability of the array were further assessed. Figure 4c,d displays the *I‐V* curves of multiple cells across the array. Except for a few outliers that remained in the HRS, the array demonstrates satisfactory device‐to‐device consistency. Figure 4e–f presents the mapping of the rectification ratio for each cell, confirming that the self‐rectifying property is uniform across the array. Figure S12a–d shows the write/read/erase protocols for both device types. For the Ag device, the read voltage is set at −0.3 V, with write and erase pulses at −0.8 and +0.8 V, respectively (polarities are reversed for the AgCl_x_/Au device). The devices can be reversibly switched between HRS and LRS, with stable cycling demonstrated over multiple operations. These results highlight the device's potential for reliable, high‐density memory storage with built‐in crosstalk suppression, paving the way for selector‐free crossbar array applications.

**FIGURE 4:**
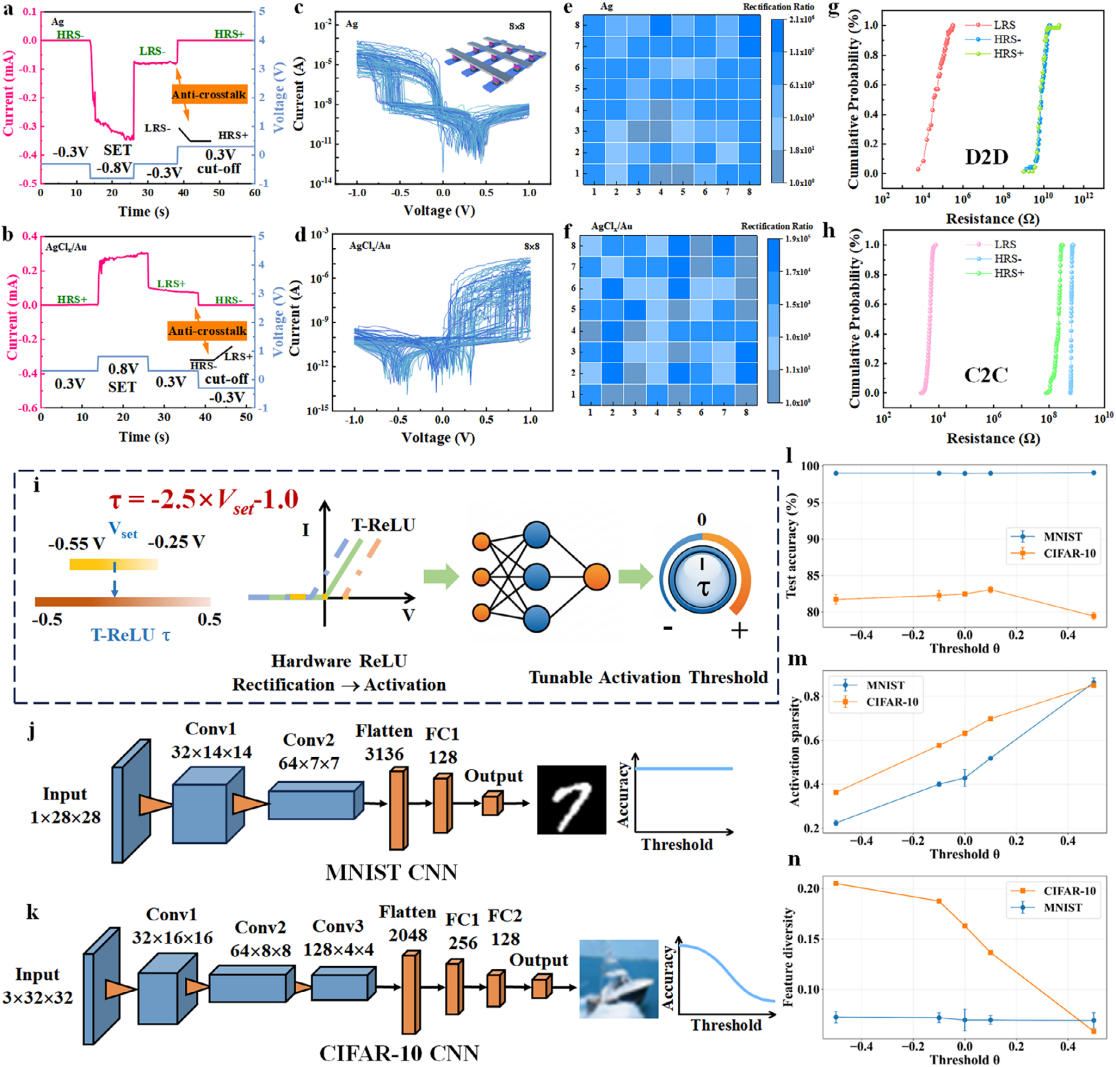
8 × 8 crossbar array performance and neural network simulation with tunable T‐ReLU activation. (a) Self‐rectifying behavior of the Ag‐electrode device, with the SET voltage (−0.8 V), read voltage (−0.3 V), and reverse‐bias cut‐off voltage (0.3 V). (b) Self‐rectifying behavior of the AgCl_x_/Au‐electrode device, with the SET voltage (0.8 V), read voltage (0.3 V), and reverse‐bias cut‐off voltage (−0.3 V). (c, d) *I‐V* uniformity of multiple cells across the array. In the 8 × 8 arrays, 52 of the 64 Ag cells and 47 of the 64 AgCl_x_/Au cells showed stable resistive switching; the *I‐V* curves of these cells are shown. (e, f) Mapping of the rectification ratio for each cell in the array. (g, h) Cumulative probability distributions of HRS and LRS currents for both D2D and C2C exhibit narrow spreads, confirming the favorable device uniformity and switching stability. (i) Schematic illustration of the T‐ReLU activation function with dynamically modulated activation threshold (τ). (j) CNN architecture for MNIST dataset. (k) CNN architecture for CIFAR‐10 dataset. Images are reproduced from the publicly available MNIST handwritten‐digit database (LeCun, Cortes & Burges, 1998; http://yann.lecun.com/exdb/mnist/) and the CIFAR‐10 dataset (Krizhevsky, 2009; https://www.cs.toronto.edu/~kriz/cifar.html). (l–n) Testing accuracy, feature sparsity, and feature diversity analysis across both architectures as a function of threshold τ. The results demonstrate that the device's tunable rectifying characteristics can be exploited for neuromorphic computing applications with potential computational savings.

To comprehensively evaluate the device uniformity and reproducibility, we statistically characterized 80 randomly selected devices from 10 independent chips (8 devices per chip). The fabrication yield was determined to be 91.25% (Figure S12e). The box‐plot statistics show that the ON/OFF ratio and rectification ratio have mean values of ∼ 2 × 10^5^ and median values of ∼ 1.5 × 10^5^, with a wide range (from ∼ 10^3^ to ∼ 10^6^) (Figure S12f). To further quantify the variability, we plotted the cumulative probability distributions of the HRS and LRS currents across different devices (D2D, Figure 4g) and across repeated switching cycles (C2C, Figure 4h), the latter of which was analyzed from over 2000 cycling tests. To evaluate the read reliability in a crossbar configuration, we characterized a fabricated 8 × 8 array with all unselected WLs/BLs left floating to eliminate parasitic leakage. The calculation procedures are detailed in Tables S3–S5. The target cells exhibited I_on_ = 5 × 10^−6^ A, I_off_ = 7 × 10^−11^ A, and I_reverse_ = 2 × 10^−11^ A at ±0.3 V (averaged over five cells; Table S4). Under the 1/3 V_read_ biasing scheme, the sneak current is 1 × 10^−10^A, and the total read current is 1 × 10^−9^A, yielding a read margin of approximately 99.98% for the 8 × 8 array, confirming effective sneak‐path suppression at the 8 × 8 scale. Extending the calculation to larger arrays, the RM remains as high as 93.55% for 128 × 128 but drops below 30% at approximately 420 × 420 and approaches 10% at around 470 × 470. The device's potential for small‐ to medium‐scale integration, while highlighting the need for optimized biasing and interconnect engineering for large‐scale applications in future work.

We further exploited the tunable rectifying characteristics (the preceding bias‐dependent *V_set_*, Figure 3m and Figure S10) of the device to map the experimentally measured threshold voltage to the T‐ReLU parameter τ for software implementation. As illustrated in Figure 4i, the experimentally measured threshold voltage directly correlates with the T‐ReLU simulation parameter. The transformation τ = −2.5 × *V_set_*‐1.0 is derived from a direct linear mapping between the experimentally accessible *V_set_* range and the simulation τ range. The *V_set_* of our device can be electrically programmed from approximately −0.6 to −0.2 V (Figure 3l,m). To map this range to the T‐ReLU threshold τ range used in our simulations (−0.5 to 0.5), we solve *V_set_* = ατ+β using the boundary conditions: *V_set_* = −0.6 V at τ = 0.5, and *V_set_* = −0.2 V at τ = −0.5. This gives α = −0.4 and β = −0.4, yielding the inverse mapping τ = −2.5 × *V_set_*‐1.0. This transformation is a numerical normalization between the measured *V_set_* range and the simulation τ range, not a physical implementation of the full T‐ReLU operation. Table S6 summarizes the complete mapping. This mapping is derived directly from experimental measurements (Figure S10), demonstrating that our device can provide the complete threshold range required for mapping the T‐ReLU parameter τ.

To this end, two distinct Convolutional Neural Network (CNN) architectures were designed and deployed on the MNIST and CIFAR‐10 datasets. Specifically, the MNIST model features a lightweight topology comprising an input layer (1 × 28 × 28), two cascaded convolutional layers (32 to 64 channels), batch normalization, T‐ReLU activation, and pooling operations, terminating in a fully connected layer (10 nodes, Figure 4j). In contrast, to accommodate the augmented complexity of natural RGB images, the CIFAR‐10 model was scaled into a deeper architecture featuring three convolutional layers (32→64→128 channels) and two fully connected layers (Figure 4k).

Figure 4l–n delineate the testing accuracy, feature sparsity, and feature diversity across both architectures. For the structurally simpler MNIST model, the testing accuracy exhibits remarkable immunity to variations in the positive threshold τ, whereas the activation sparsity scales monotonically. This indicates that for relatively simple image‐classification tasks like MNIST recognition, imposing a large τ (τ = 0.5) serves as an optimal strategy, driving high activation sparsity without incurring accuracy degradation. Conversely, the more complex CIFAR‐10 model exhibits a distinct inverse dependence: while enlarging τ successfully induces higher sparsity, it precipitates a noticeable degradation in accuracy. Essentially, aggressive thresholding erodes feature diversity, and this erosion is catastrophic for complex data (CIFAR‐10) processed by deep networks, but inconsequential for simple data (MNIST) handled by shallow ones. To systematically exploit these threshold‐dependent dynamics, we subsequently partitioned our investigation into two distinct pathways: unlocking the potential of negative thresholds for sensory enhancement, and harnessing positive thresholds for potential computational savings.

By operating in the negative threshold regime (τ < 0), the activation barrier is lowered so that weak sub‐threshold activations are admitted. As depicted in Figure 5a, we evaluated this using a customized architecture for nighttime recognition. Figure 5b shows that a negative threshold maintains recognition accuracy comparable to the ReLU baseline (91.0% at τ = −0.5 vs. 90.9% at τ = 0), whereas positive thresholds degrade sharply (85.4% at τ = +0.1, collapsing to 44.9% at τ = +0.5). Thus, in photon‐starved low‐light scenes where weak signals must not be discarded, the network should operate at the negative/zero end of the threshold range rather than the positive end. Because the negative threshold is mathematically equivalent to a positive bias shift, it can be implemented directly by the device's non‐volatile, electrically programmable *V_set_* without additional biasing circuitry. Comparison of activation functions for low‐light recognition is shown in Table S8. The confusion matrices in Figure 5c and Figure S13 show that the negative threshold yields a misclassification pattern comparable to the τ = 0 baseline, confirming that it maintains recognition capability without the degradation observed at positive thresholds.

**FIGURE 5:**
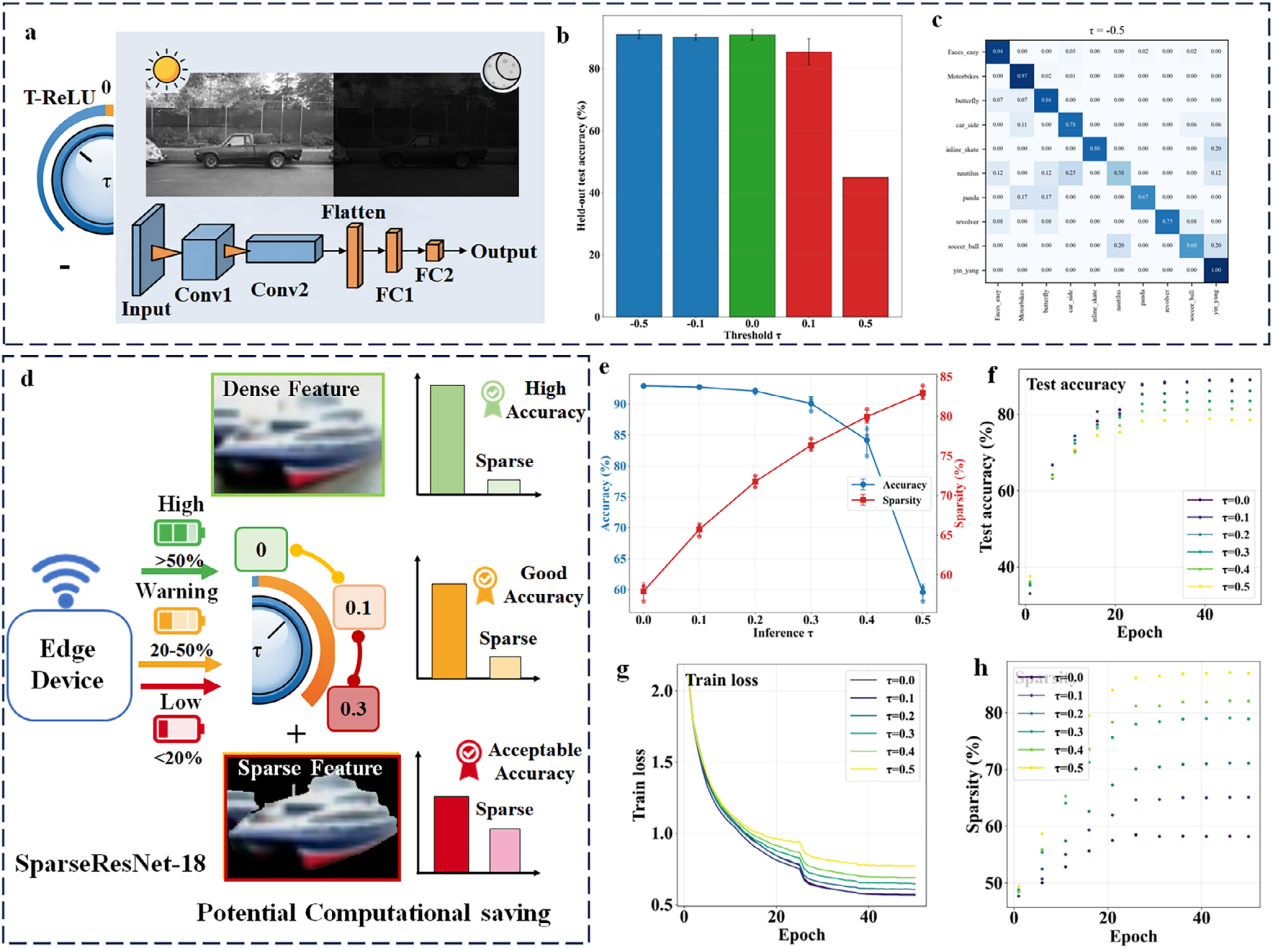
Neuromorphic computing performance with tunable T‐ReLU thresholds for low‐light enhancement and sparsity‐aware edge computing. (a) Customized CNN architecture designed for nighttime recognition tasks. Images are reproduced from the publicly available Caltech 101 dataset (Li et al., 2022; https://doi.org/10.22002/D1.20086) and subsequently edited via Python scripts to construct our custom “night” dataset. (b) Testing accuracy comparison for negative threshold configurations (τ = −0.5 to 0.5), showing accuracy comparable to the ReLU baseline. (c) Confusion matrix for τ = −0.5 (held‐out test set). (d) Schematic of the battery‐level‐aware modulation paradigm, switching between high‐accuracy (high‐battery) and high‐sparsity (low‐battery) modes. Images are reproduced from the CIFAR‐10 dataset (Krizhevsky, 2009; https://www.cs.toronto.edu/~kriz/cifar.html). (e–h) Single‐checkpoint inference‐time threshold sweep (test accuracy and activation sparsity as a function of the inference threshold) over 50 epochs, quantitatively validating the trade‐off between activation sparsity and inference accuracy.

Recalling our earlier observation, the simple CNN architecture suffered from severe accuracy degradation on the complex CIFAR‐10 dataset when subjected to large positive thresholds. To overcome this representational bottleneck and practically harness the positive threshold regime (τ > 0) for dynamic edge computing with potential computational savings, a more resilient architectural backbone was fundamentally mandated. Consequently, we transitioned from the simple CNN to a deeper ResNet‐18 framework. As schematized in Figure 5d, we propose a battery‐level‐aware modulation paradigm: During high‐battery mode, τ is minimized (τ = 0) to retain granular feature details and guarantee supreme accuracy; during low‐battery mode, the simulated network switches to a high τ during inference to suppress low‐confidence features. This generates an exceedingly high proportion of zero‐valued activations, which could reduce MAC operations if zero‐skipping is supported by the underlying hardware.

To instantiate this paradigm, we engineered a lightweight, dynamically sparse architecture (dynamic sparse edge‐net via T‐ReLU) based on a highly customized ResNet‐18 (Figure S14). To prevent the aforementioned residual noise, we implemented an “anti‐residual interference” modification, mandating that fused features from shortcut connections undergo a strict secondary T‐ReLU filtration (Figure S15). Furthermore, to protect fragile low‐level visual information, a “First‐Layer Exemption” mechanism was enforced, permanently locking the stem layer's threshold at τ = 0. Directly applying a high positive threshold (τ > 0) from the initial epoch causes a catastrophic “parabolic collapse” in testing accuracy (as shown in Figure S16). To mitigate this, we introduced a warm‐up strategy that allows the network to adaptively restructure its weight distribution before reaching the target sparsity. We trained a single SparseResNet‐18 (τ = 0, 50 epochs) and swept the threshold τ at inference without any weight update (Figure 5e–h) to demonstrate that the battery‐aware modulation requires no retraining. For τ∈[0, 0.3], the network exhibits a graceful trade‐off: activation sparsity rises from 57.9% to 76.3% while accuracy decreases only from 93.0% to 90.1%. Beyond τ ≈ 0.3 the degradation steepens (84.2% at τ = 0.4), and the single network collapses at τ = 0.5 (59.6%). The full range up to τ = 0.5 (85.97% accuracy, 86.8% sparsity) is instead reached by per‐threshold‐trained models (Figure S17). All neural‐network results are obtained from PyTorch simulations using the experimentally measured *V_set_* to τ mapping. A full hardware integration, in which weights are mapped onto a memristive crossbar array, and the threshold element is integrated with peripheral circuits, remains an important direction for future research.

## 3 Conclusion

In summary, we have successfully demonstrated a high‐performance, self‐rectifying CsCuCl_3_‐based memristor with dynamically tunable threshold characteristics. The switching mechanism is governed by the synergistic action of two processes: bulk ion migration within CsCuCl_3_, which provides and sustains the low‐resistance state essential for achieving a high ON/OFF ratio, and the AgCl_x_ interfacial layer, which introduces a Schottky barrier and trap filling/release mechanism that controls the actual resistive switching. Together, these mechanisms enable the device to achieve an ON/OFF ratio exceeding 10^5^ and a rectification ratio greater than 10^4^, along with robust retention and endurance performance. Moreover, the self‐rectifying property enabled effective sneak path current suppression in an 8×8 crossbar array without requiring external selector devices. Systematic simulations using CNN architectures quantified a dataset‐dependent threshold sensitivity: MNIST accuracy remained robust regardless of threshold variations, whereas CIFAR‐10 accuracy dropped sharply as positive thresholds increased. Based on this trend, we exploited the bi‐directional tunability for dual‐mode applications: (1) Negative threshold operation in CNNs maintained 91.0% held‐out test accuracy for low‐light image recognition (on par with ReLU), avoiding the accuracy collapse of positive thresholds. (2) Positive threshold modulation in a customized SparseResNet‐18 architecture enabled sparsity‐aware edge computing. To ensure robustness, we implemented architectural modifications including anti‐residual interference, first‐layer exemption, and a warm‐up strategy. As the threshold scaled from 0.0 to 0.3, activation sparsity surged from 57.9% to 76.3%, accompanied by a controlled accuracy degradation from 93.0% to 90.1% (single network, no retraining). We aspire, through the advancement of this work, to significantly enhance the unit density and agility of information computation and storage, thereby providing robust support for overcoming various challenges in the field of information intelligence.

## 4 Experimental Section

### 4.1 Synthetic CsCuCl_3_

Equimolar amounts of CsCl (99.9%, Macklin) and CuCl_2_ (99.99%, Macklin) powders were dissolved in 1 mL of a mixed solvent containing DMSO (99.9%, Aladdin) and DMF (99.999%, Aladdin) (95:5, v/v). Subsequently, 0.05 g of BMIMBF_4_ (97%, Macklin) was added to the mixture. The system was magnetically stirred at 70°C for 12 h. After stirring, the mixture was filtered through a 0.22 µm PTFE syringe filter to obtain the brownish‐yellow saturated CsCuCl3 precursor solution.

### 4.2 Device Fabrication

Patterned ITO glass substrates were sequentially cleaned in an ultrasonic bath with deionized water, acetone, deionized water, and anhydrous ethanol for 15 min each. The cleaned substrates were dried under a steady nitrogen stream and treated with plasma for 180 s to improve surface wettability. A diluted SnO_2_ aqueous solution (deionized water to 15 wt.% SnO_2_ colloidal dispersion volume ratio of 4:1) was spin‐coated onto the ITO substrates at 4000 rpm for 30 s. The substrates were then thermally annealed in ambient air at 150°C for 30 min, followed by a 30 min UVO treatment. The prepared ITO/SnO_2_ substrates and the filtered CsCuCl_3_ precursor solution were preheated on a hot plate at 75°C. Inside a nitrogen‐filled glovebox, 50 µL of the preheated CsCuCl_3_ precursor was spin‐coated onto the substrate at 3500 rpm for 30 s. At the 10th second of the spin‐coating process, IPA (99.99%, Aladdin) was swiftly dropped onto the center of the spinning substrate as an anti‐solvent. The as‐spun films were then annealed at 120°C for 10 min to promote crystallization. Ultimately, a 100 nm‐thick top metal electrode (Au, Ag, Cu, or Al) was deposited onto the CsCuCl_3_ film via electron beam evaporation through a shadow mask, completing the ITO/SnO_x_/CsCuCl_3_/Metal memristor structure.

### 4.3 Fabrication of the 8 × 8 Arrays

Patterned ITO substrates (15 × 15 mm^2^) were purchased from Advanced Electron Technology Co., Ltd. The substrates consist of parallel ITO bottom electrodes with dimensions of 14 × 1 mm^2^ and an inter‐electrode spacing of 1 mm. The functional layers were deposited using the identical process parameters described for the single devices. Subsequently, top electrodes (14 × 1 mm^2^) were deposited perpendicular to the bottom ITO stripes with a 1 mm spacing between adjacent lines. This orthogonal configuration yielded an effective active area of 1 × 1 mm^2^ for each memory cell in the crossbar array.

### 4.4 Interface and Buried Interface Delamination Treatment

To expose the interface between CsCuCl_3_ and the metal electrode, a physical delamination method was applied. Adhesive tape was pressed firmly onto the device surface and then swiftly peeled away, revealing the CsCuCl_3_/metal electrode interface for further characterization and analysis.

### 4.5 Measurement

The crystallographic structure and phase composition of the samples were determined by x‐ray diffraction (XRD) using Cu(Ka) radiation. X‐ray photoelectron spectroscopy (XPS) was performed on an Axis Supra+ system (Shimadzu Corporation) using an Al x‐ray source, and all binding energies were carefully calibrated against the adventitious C 1s peak at 284.8 eV. The optical absorption properties were evaluated using a UV–vis–NIR spectrophotometer. To elucidate the energy band structure of the perovskite films, ultraviolet photoelectron spectroscopy (UPS) was employed to extract the work function and the valence band maximum (VBM). Capacitance‐frequency (C‐f) measurements were recorded on an electrochemical workstation (AUTOLAB). For temperature‐dependent C‐f profiling, the device temperature was precisely regulated using a liquid nitrogen controller (ST‐100, Physike Technology Co., Ltd.). Surface potential mapping was conducted via Kelvin probe force microscopy (KPFM, Bruker Icon) equipped with Pt/Ir‐coated conductive probes (SCM‐PIT‐V2). The elemental depth profiles were characterized by TOF‐SIMS (TOF.SIMS 5, IONTOF GmbH, Germany) using a 2 keV O sputtering beam and a 30 keV Bi_3_^+^ analysis beam. The sputtering and analysis areas were 300 × 300 µm^2^ and 100 × 100 µm^2^, respectively.

All electrical characterizations were carried out utilizing a Keithley 2636B source meter. A 10 mA current compliance was enforced during voltage sweeps to prevent hard breakdown. For stability measurements, devices were stored under ambient air at room temperature (approximately 25°C) with 40%–60% relative humidity without encapsulation, and devices were kept in a cleanroom environment with no additional encapsulation throughout testing. The ON/OFF ratio corresponds to the ratio of currents read at −0.3 V for the LRS and HRS, respectively. The LRS current was acquired following a negative voltage sweep up to −1 V, where a 10 mA compliance current was set to avoid hard breakdown, whereas the HRS current was obtained at −0.3 V in the subsequent positive sweep after device reset. The rectification ratio (RR) is given by RR = |I_LRS_(‐0.3 V)| / |I_HRS_(+0.3 V)|, calculated as the ratio of the forward current I_forward_ at −0.3 V under the LRS state to the reverse current I_reverse_ at +0.3 V under the HRS state. All *I‐V* sweeps were executed at a sweep rate of 0.1 V/s. Unless otherwise specified (e.g., the frequency‐dependent measurements in Figure 3j), all *I‐V* sweeps were performed at a sweep rate of 0.1 V/s. For retention, the device was first programmed to the target resistance state (HRS or LRS) using the appropriate SET or RESET pulse. The current was then monitored at a fixed read voltage of −0.3 V for the LRS and HRS in the negative bias region, and at +0.3 V for the HRS in the positive bias region. For endurance, the device was subjected to repeated write‐read‐erase‐read cycles. The SET operation was performed using a −0.8 V pulse with a width of 200 ms, and the RESET operation was performed using a +0.8 V pulse with a width of 200 ms. After each write or erase pulse, the resistance state was read at −0.3 V for the LRS and HRS in the negative region, and at +0.3 V for the HRS in the positive region. For crossbar array measurements, the selected ITO and Ag lines were connected to the Keithley 2636B, while all other lines were left floating. The read‐margin calculation was performed using the measured single‐cell parameters under a 1/3 Vread biasing scheme.

### 4.6 Neural Network Simulation and Architectures

The neuromorphic computing performance was simulated using the PyTorch framework. The memristive behavior was modeled using a custom Threshold‐ReLU (T‐ReLU) activation function, defined as F(x) = x for x>τ; F(x) = 0 for x≤τ;, where τ represents the tunable threshold voltage derived from the experimental* V_set_* characteristics. Three distinct neural network architectures were deployed to evaluate device performance across different complexity levels:

i. MNIST CNN: A simple convolutional neural network designed for grayscale digit recognition (28 × 28 input). The architecture comprises an input layer, two cascaded convolutional layers (32→64 channels), batch normalization, T‐ReLU activation, max pooling operations, and a fully connected classification layer (10 nodes). This model was used to evaluate basic threshold tunability and low‐light enhancement capabilities.
ii. CIFAR‐10 CNN: A deeper convolutional network designed for RGB image recognition (32 × 32 × 3 input). This model features three convolutional layers (32→64→128 channels) and two fully connected layers. It was employed to investigate the trade‐off between accuracy and sparsity under positive threshold modulation.
iii. A customized ResNet‐18 framework (CIFAR‐10 SparseResNet‐18) was designed for robust sparsity‐aware edge computing. The network accepts 3 × 32 × 32 RGB inputs and consists of a Stem layer with Conv2d (3 × 3), BatchNorm, and T‐ReLU activation producing 64 channels, followed by four residual layers (Layer 1‐4) composed of SparseBasicBlocks. Layer 1 maintains feature dimensions while Layers 2‐4 incorporate downsampling blocks to progressively reduce spatial dimensions and increase channel depth to 128, 256, and 512, respectively, terminating in a classification head with adaptive average pooling and a fully connected layer (10 nodes). To optimize performance, the “First‐Layer Exemption” mechanism permanently locks the Stem layer threshold at τ = 0 to preserve low‐level visual features, while the “Anti‐Residual Interference” modification mandates secondary T‐ReLU filtration for fused features from shortcut connections to prevent residual noise. Furthermore, a “Warm‐up Strategy” was employed during training to allow adaptive weight restructuring before applying high positive thresholds, preventing accuracy collapse, complemented by a gradual threshold ramping strategy to ensure stability during high‐sparsity training. The complete training protocol is shown in Table S7.

All networks were trained using the stochastic gradient descent (SGD) optimizer. Activation sparsity was calculated as the percentage of zero‐valued outputs after T‐ReLU activation to estimate potential computational savings. Actual energy savings depend on hardware support for zero‐skipping or sparse computation. For the SparseResNet‐18 model, a gradual threshold ramping strategy was implemented to avoid accuracy collapse during high‐sparsity training. We used Gemini 3.1 Pro to assist with code generation and programming support for the implementation of the proposed method. We reviewed and edited the code thoroughly and take full responsibility for the content of the published article.

## Author Contributions

**Qingwei Shi**: methodology, formal analysis, visualization, data curation, Writing – original draft. **Mufan Zhu**: methodology, data curation, formal analysis, visualization. **Song He**: validation, investigation, visualization. **Zhen Yan**: software, formal analysis, visualization. **Yuezhong Lu**: formal analysis, investigation, validation. **Shuaidi Wu**: formal analysis, validation, investigation. **Wenju Xie**: methodology, formal analysis. **Xiaofeng Wang**: methodology, formal analysis, funding acquisition. **Baochang Cheng**: funding acquisition, supervision, resources, project administration. **Jie Zhao**: writing – original draft, funding acquisition, conceptualization, project administration, writing – review and editing.

## Funding

This work was supported by the National Natural Science Foundation of China (Nos. 62464011, 62474084, 52462025) and the Natural Science Foundation of Jiangxi Province (Nos. 20242BAB25226, 20242BAB25097).

## Conflicts of Interest

The authors declare no conflicts of interest.

## Supporting information




**Supporting File 1**: advs78702‐sup‐0001‐SuppMat.docx.

**Supporting File 2**: advs78702‐sup‐0002‐SuppMat.zip.

## Data Availability

The data that support the findings of this study are available from the corresponding author upon reasonable request.
