# Supplementary material for: Interface‐Mediated Self‐Rectifying CsCuCl3 Memristors With Tunable Thresholds for Reconfigurable Neuromorphic Computing
Source: Adv Sci (Weinh). 2026 Nov 9:e78702. Online ahead of print. doi: 10.1002/advs.78702 (PMC13748775; doi:10.1002/advs.78702)
Supplement: Supplementary file 1 [file ADVS-9999-e78702-s001.docx]

Supporting Information

Interface-Mediated Self-Rectifying CsCuCl_3_ Memristors with Tunable Thresholds for Reconfigurable Neuromorphic Computing

Qingwei Shi, Mufan Zhu, Song He, Zhen Yan, Yuezhong Lu, Shuaidi Wu, Wenju Xie, Xiaofeng Wang, Baochang Cheng* and Jie Zhao*

**Figure S1.** SEM images and corresponding EDS elemental mappings of CsCuCl_3_ films prepared under different conditions. a-d) target film with both additive BMIMBF_4_ and antisolvent IPA, e-h) film without BMIMBF_4_, i-l) film without antisolvent IPA treatment.

**Figure S2.** a) XPS survey spectrum of CsCuCl_3_. b) C1s core-level spectrum used for charge correction.

**Figure S3.** SET voltage statistics of the ITO/SnO_2_/CsCuCl_3_/Ag/Au device over 200 cycles.

**Figure S4.** a) High-resolution Cl 2p XPS spectra of the CsCuCl₃ interface under three conditions: pristine (0 V), after 1 V stressing (close to the normal SET/RESET voltage of ±0.8 V), and after 5 V accelerated stressing. b-c) High-resolution Ag 3d and Au 4f XPS spectra of the CsCuCl_3_ interface before and after electrical stressing (0 V vs 5 V), showing enhanced Ag oxidation. d-e) Cu 2p XPS spectra of the CsCuCl_3_ layer under Ag and Au electrodes.

**Figure S5.** a, b) XPS survey spectra of the CsCuCl_3_/Ag interface under 0 V and 5 V stimulation. c, d) CsCuCl_3_/Au interface under 0 V and 5 V stimulation.

**Figure S6.** ToF-SIMS reconstructions of the ITO/SnO_x_/CsCuCl_3_/Ag stack, showing the spatial distribution of Ag^+^, AgCl^+^, Cs^+^, Cu^+^, and Sn^+^ ions as a function of sputter time.

**Figure S7.** *V_set_* distribution under different voltage sweep amplitudes.

**Figure S8.** Schematic illustration of the carrier transport mechanism for the AgCl_x_/Au electrode device.

**Figure S9.** KPFM surface potential mapping and temperature-dependent I-V (50-75 ^o^C). a) KPFM image of the half-covered CsCuCl_3_/Ag sample. b) CPD line-scan across the CsCuCl_3_/Ag junction. c) KPFM image of the half-covered CsCuCl_3_/AgCl_x_/Au sample. d) CPD line-scan across the CsCuCl_3_/AgCl_x_/Au junction. e) Temperature-dependent I-V curves from 50 °C to 75 °C. f) Corresponding linear fitting of ln *I* versus *V* in the forward-bias region.

**Figure S10.** Nonvolatile retention, drift, cycle-to-cycle variability, device-to-device reproducibility, and programming endurance of electrically programmed threshold states. a-e) Nonvolatile retention of the programmed resistance state. The device is programmed to different *V_set_* values by preceding positive biases of varying amplitudes: 0 V, 0.1 V, 0.2 V, 0.4 V, and 0.6 V. Switching to the LRS occurs only when the applied negative voltage exceeds the programmed threshold *V_set_*, demonstrating that each programmed state corresponds to a distinct and stable T-ReLU threshold setting. After set process, the device remains stable LRS over 1000 s, confirming the nonvolatile nature of the programmed resistance state. The corresponding τ values are labeled in the upper left corner. f) Retention and drift of the programmed threshold states over 1000 s. g) *V_set_* versus cycle number for five programmed threshold states over 20 cycles each. h) Cumulative probability distributions of *V_set_* for the five programmed states, illustrating cycle-to-cycle variability. i) Device-to-device reproducibility of *V_set_*. j) statistical distribution of *V_set_* across multiple devices.

The Schottky barrier height was extracted using the thermionic emission (TE) model, following standard procedures established in the literature.

1. Single-Temperature Fitting

For each temperature, the forward-bias I-V characteristics were analyzed using the TE equation:

$$I=I_{0}\left[ \exp\left( \frac{qV}{nkT} \right)-1 \right]$$

In the linear region (where *V*>3*kT*), this equation can be rewritten as:

$$\ln I=\ln I_{0}+\frac{q}{nkT}\cdot V$$

For each temperature, ln(*I*) was plotted against *V*, and a linear fit was performed in the region where thermionic emission dominates. From the linear fit:

Slope $S=q/(nkT)$ → Ideality factor: $n=q/(S\cdot k\cdot T)$

Intercept $C=\ln I_{0}$ → Reverse saturation current: $I_{0}=\exp(C)$

2. Richardson Plot Analysis

The temperature-dependent *I_0_* values were then used to construct the Richardson plot based on the equation:

$$\ln\left( \frac{I_{0}}{T^{2}} \right)=\ln(AA^{*})-\frac{q\Phi_{B}}{k}\cdot\frac{1}{T}$$

A linear fit of $\ln(I_{0}/T^{2})$ versus $1000/T$ yields a slope S_R_, and the effective Schottky barrier height is extracted from:

$$\Phi_{B}=-\frac{S_{R}\times1000\times k}{q}$$

where *k* is the Boltzmann constant (8.617×10^-5^ eV/K) and *q* is the elementary charge (1.602×10^-19^ C). The Richardson constant *A** was taken as the theoretical value of 120 A/cm^2^K^2^.

**Table** **S1.** Summary of temperature-dependent I-V fitting parameters and extracted Schottky barrier heights.

| T | 1000/T | S | C | n | I_0_ (A) | ln(I_0_/T^2^) | ln(AA*T^2^) | Φ (eV) |
| --- | --- | --- | --- | --- | --- | --- | --- | --- |
| 50°C | 3.095 | 3.49 | 23.75 | 1.03 | 2.06×10^10^ | 14.80 | 14.23 | 0.018 |
| 60°C | 3.002 | 3.47 | 22.49 | 1.00 | 5.83×10^9^ | 13.64 | 14.31 | 0.030 |
| 75°C | 2.872 | 3.52 | 22.00 | 0.95 | 3.58×10^9^ | 12.89 | 14.43 | 0.058 |

**Table S2.** Fitting parameters of the Richardson plot in the temperature range of 50-75 °C

| Parameters | Value |
| --- | --- |
| Slope | -5.31 |
| R^2^ | 0.993 |
| Effective barrier height | Φ_B_ = 0.46 eV |

**Figure S11.** Optical images of 8×8 crossbar arrays comprising both Ag and AgCl_x_/Au memristors.

**Figure S12.** a-b) Non-volatile memory with stable cycling demonstrated over multiple operations. c-d) Schematic of the write/read/erase protocols for Ag and AgCl_x_/Au devices, demonstrating non-volatile memory operation. e) Device yield for 80 randomly selected devices from 10 independent chips (8 devices per chip), showing an overall yield of 91.25%. f) Distributions of ON/OFF ratio and rectification ratio extracted from D2D measurements across 80 devices.

**Theoretical Evaluation of Crossbar Array**

We first performed an ideal estimation using the measured single-device parameters. The read voltage was set at V_read_= 0.2 V (for I_reverse_) or -0.2 V (for I_on_ and I_off_). Based on the typical single-device characteristics, the ON-state current is I_on_= 5×10^-5^A, the OFF-state current is I_off_ = 6×10^-11^A, and the reverse leakage current is I_reverse_= 4×10^-11^A. In an 8×8 array under the worst-case condition (target cell OFF, all other cells ON), the sneak-path current can be estimated as:

I_sneak_≈ (N-1)×(N-1) × I_reverse_

The total read current when the target cell is OFF:

I_read_off_= I_off_ + I_sneak_

The read margin (RM) is then calculated as:

RM = (I_on_- I_read_off_)/ I_on_

The theoretical maximum array size under different RM criteria is summarized in Table S3.

**Table S3.** Theoretical maximum array size under different read margin criteria.

| Array size | I_sneak_ (A) | I_read_ (A) | RM |
| --- | --- | --- | --- |
| 8×8 | 2.0×10^-9^ | 2.0×10^-9^ | 99.99% |
| 16×16 | 9.0×10^-9^ | 9.1×10^-9^ | 99.98% |
| 32×32 | 38.4×10^-9^ | 38.5×10^-9^ | 99.92% |
| 64×64 | 158.8×10^-9^ | 158.8×10^-9^ | 99.68% |
| 128×128 | 645.2×10^-9^ | 645.2×10^-9^ | 98.71% |
| 256×256 | 2.6×10^-6^ | 2.6×10^-6^ | 94.80% |
| 512×512 | 10.4×10^-6^ | 10.4×10^-6^ | 79.12% |
| 1,024×1,024 | 41.9×10^-6^ | 41.9×10^-6^ | 16.28% |

When integrated into the 8×8 crossbar array, the ON-state current of the target cells is I_on_= 5×10^-6^A, while the OFF-state current I_off_ = 7×10^-11^A, and the reverse leakage current is I_reverse_= 2×10^-11^A (extracted from five cells; see Table S4 for detail). To extract the intrinsic device parameters within the array environment, we performed measurements with all unselected word lines (WLs) and bit lines (BLs) left floating, eliminating parasitic leakage paths. Based on this measured parameter, we further simulated the sneak-path scenario under the standard 1/3 V_read_ biasing scheme, where all 49 unselected cells are assumed to be reverse-biased (-1/3 V_read_). To evaluate the scalability of the proposed device, we extended this calculation to various array sizes (Table S5). The sneak current is 1×10^-10^A, and the total read current is 1×10^-9^A, yielding a read margin of approximately 99.98% for the 8×8 array. The read margin remains as high as 93.55% for a 128×128 array but degrades with further scaling: it drops below 30% at approximately 420×420 and approaches 10% at around 470×470, beyond which reliable reading cannot be guaranteed. The proposed device can suppress sneak-path interference at the prototype level, supporting the core concept of this work. However, the validated read range is currently limited to small-scale configurations. Future work will focus on combining optimized fabrication processes with improved read biasing strategies to extend the device feasibility to large-scale, high-density crossbar arrays.

**Table S4.** Parameters extracted from five target cells

|  | Cell 1 (A) | Cell 2 (A) | Cell 3 (A) | Cell 4 (A) | Cell 5 (A) | Average (A) |
| --- | --- | --- | --- | --- | --- | --- |
| I_on_ | 7×10^-6^ | 3×10^-6^ | 2×10^-6^ | 8×10^-6^ | 6×10^-6^ | 5×10^-6^ |
| I_off_ | 2×10^-10^ | 2×10^-11^ | 1×10^-10^ | 3×10^-11^ | 1×10^-11^ | 7×10^-11^ |
| I_reverse_ | 3×10^-11^ | 1×10^-11^ | 6×10^-12^ | 2×10^-11^ | 2×10^-11^ | 2×10^-11^ |

**Table S5.** Calculated read margin (RM) and sneak current (I_sneak_) for various crossbar array sizes, based on the measured array-level parameters

| Array size | I_sneak_ (A) | I_read_ (A) | RM |
| --- | --- | --- | --- |
| 8×8 | 1.0×10^-9^ | 1.1×10^-9^ | 99.98% |
| 16×16 | 4.5×10^-9^ | 4.8×10^-9^ | 99.91% |
| 32×32 | 19.2×10^-9^ | 19.3×10^-9^ | 99.61% |
| 64×64 | 79.4×10^-9^ | 79.5×10^-9^ | 98.41% |
| 128×128 | 322.6×10^-9^ | 322.7×10^-9^ | 93.55% |
| 256×256 | 1.3×10^-6^ | 1.3×10^-6^ | 74.00% |
| 420×420 | 3.5×10^-6^ | 3.5×10^-6^ | 29.8% |
| 470×470 | 4.4×10^-6^ | 4.4×10^-6^ | 12.0% |
| 512×512 | 5.2×10^-6^ | 5.2×10^-6^ | -4.4% |

**V_set_-to-τ mapping and hardware validation.**

To directly correlate the hardware programmable threshold with the T-ReLU activation function, we established a linear mapping between the experimentally measured threshold voltage V_set_ and the simulation parameter τ:

$$\tau=-2.5\times V_{set}-1.0$$

This transformation maps the hardware V_set_ range (-0.6 V to -0.2 V) to the simulation τ range (-0.5 to +0.5), while simultaneously performing polarity reversal (negative V_set_ maps to positive τ). The resulting τ values are further discretized to 0.1-step intervals using upward rounding (e.g., 0.11→0.2, 0.32→0.4) to match the discrete threshold settings used in simulations. Table S6 summarizes the complete V_set_-τ mapping. The data processing routine calculates the corresponding τ values. The nonvolatile threshold programming characteristics of the device are validated in Figure S8.

**Table S6.** Mapping between hardware threshold voltage $V_{set}$ and discretized simulation parameter τ. The mapping is given by $\tau=-2.5\times V_{set}-1.0$, followed by 0.1-step upward rounding discretization.

| V_set_ | τ | τ_rep |
| --- | --- | --- |
| -0.20 V ~ -0.22 V | τ ≤ -0.5 | -0.5 |
| -0.24 V ~ -0.26 V | (-0.5, -0.4] | -0.4 |
| -0.28 V ~ -0.30 V | (-0.4, -0.3] | -0.3 |
| -0.32 V ~ -0.34 V | (-0.3, -0.2] | -0.2 |
| -0.36 V ~ -0.38 V | (-0.2, -0.1] | -0.1 |
| -0.40 V | (-0.1, 0) | 0 |
| -0.42 V ~ -0.44 V | [0, 0.1) | 0.1 |
| -0.46 V ~ -0.48 V | [0.1, 0.2) | 0.2 |
| -0.50 V ~ -0.52 V | [0.2, 0.3) | 0.3 |
| -0.54 V ~ -0.56 V | [0.3, 0.4) | 0.4 |
| -0.58 V ~ -0.60 V | [0.4, 0.5] | 0.5 |

**Figure S13.** Confusion matrices for nighttime recognition at τ=0 and -0.1, providing a baseline comparison for Fig. 5c.

**Figure S14.** Architecture of the Dynamic Sparse Edge-Net based on the customized ResNet-18 framework with T-ReLU activation.

**Figure S15.** Schematic of the "anti-residual interference" modification, which mandates secondary T-ReLU filtration for fused features from shortcut connections to prevent residual noise.

**Figure S16.** Testing accuracy collapse observed when applying high positive thresholds (τ>0) from the initial epoch without the warm-up strategy, demonstrating the necessity of adaptive weight restructuring.

**Figure S17.** The full range by per-threshold-trained models

**Table S7.** Training protocol

| Hyperparameter | MNIST CNN | CIFAR-10 CNN | Low-light CNN | SparseResNet-18 |
| --- | --- | --- | --- | --- |
| Optimizer | Adam | Adam | Adam | SGD |
| Learning rate | 0.001 | 0.001 | 0.001 | 0.1 |
| Momentum | — | — | — | 0.9 |
| Weight decay | 0 | 1.00E-04 | 0 | 1.00E-04 |
| Batch size | 128 | 128 | 64 | 128 |
| Epochs | 10 | 20 | 50 | 50 |
| LR schedule | none | none | none | MultiStepLR(50%/75%, γ=0.1) |
| Data augmentation | none | RandomCrop(32,pad4)+HFlip | none | RandomCrop(32,pad4)+HFlip |
| Loss | CE | CE | CE | CE + label smoothing (0.1) |
| Train/test partition | 60k / 10k (official) | 50k / 10k (official) | stratified 70/15/15 | 50k / 10k (official) |
| Random seeds | 42/123/2024 | 42/123/2024 | 42/123/2024 | 42/123/2024 |
| Model selection | final epoch | final epoch | best validation epoch | final epoch |

**Table S8.** Comparison of activation functions for low-light recognition. All models were trained under the same protocol with a stratified 70/15/15 train/validation/test split. Results are reported as mean ± standard deviation over three independent runs.

| **Activation** | **Accuracy (%)** |
| --- | --- |
| T-ReLU τ=-0.5 (this work) | 91.01 ± 1.35 |
| Conventional ReLU (τ=0) | 90.89 ± 1.69 |
| ReLU + bias +0.5 | 84.77 ± 1.42 |
| LeakyReLU(0.1) | 90.76 ± 1.20 |
| PReLU | 90.39 ± 1.77 |
| Learnable threshold | 90.89 ± 1.69 |
